# The LINC00852/miR-29a-3p/JARID2 axis regulates the proliferation and invasion of prostate cancer cell

**DOI:** 10.1186/s12885-022-10263-6

**Published:** 2022-12-05

**Authors:** Hao Zhang, Yang Du, Peng Xin, Xiaojun Man

**Affiliations:** 1 Department of Urology, The First Hospital of China Medical University, 155 North Nanjing Street, 110001 Shenyang, Liaoning China; 2 Institute of Urology, China Medical University, Liaoning Shenyang, China

**Keywords:** LINC00852, miR-29a-3p, JARID2, Prostate cancer, Proliferation, Invasion

## Abstract

Long intergenic non-coding RNA 00852 (LINC00852) has been shown to promote the progression of many different cancers including prostate cancer. However, the involved mechanism in promoting the proliferation, migration and invasion of prostate cancer cells has not been reported. In this study, we found that LINC00852 was highly expressed in the tissue of prostate cancer using quantitative reverse transcription PCR (qRT-PCR). CCK-8 assay, colony formation experiment, Transwell migration and invasion experiments were performed to prove that the up-regulation of LINC00852 could promote the proliferation, migration and invasion of prostate cancer cells in vitro. Xenograft tumors experiments in nude mice confirmed that up-regulation of LINC00852 promoted the proliferation of prostate cancer cells in vivo. Bioinformatics predictions and dual-luciferase reporter gene assay showed that miR-29a-3p binds to the 3’-untranslated region of JARID2, and the enhancement of miR-29a-3p could reverse the effect of LINC00852 overexpression in vitro. Moreover, the results of qRT-PCR and western blot showed that LINC00852 could regulate the expression of JARID2 through miR-29a-3p induction. In summary, we demonstrated that LINC00852 played a key role in promoting the prostate cancer, and LINC00852/miR-29a-3p/JARID2 axis could be used as a target for prostate cancer treatment.

**Supplementary Information:**

The online version contains supplementary material available at 10.1186/s12885-022-10263-6.

## Introduction

Prostate cancer is the most common cancer among men, and the second leading cause of cancer death in men in the United States [1]. Although localized prostate cancer has a high long-term survival rate, metastatic disease remains the leading cause of its related death [2]. Therefore, it is necessary to investigate the pathological metastasis and progression of prostate cancer.

Long noncoding RNAs (LncRNAs) are a group of non-coding RNAs larger than 200 nt, which play a key role in regulating chromatin dynamics, gene expression, cell growth, differentiation and development. Accumulating studies have reported that LncRNAs could affect various aspects of tumorigenesis by regulating cell signaling pathways, and then affected in the occurrence, development and metastasis of cancer [3]. Luan et al. showed that LncRNA XLOC_006390 could perform as competing endogenous RNA (ceRNA) to negatively regulate the levels of miR-331-3p and miR-338-3p as a molecular “sponge”, then regulated the target genes of NRP2, EYA2 and PKM2, and promoted the occurrence and metastasis of cervical cancer [4]. Moreover, Wang et al. showed that lncRNA-HOXD-AS1 was highly expressed in HCC tissues and related to the poor prognosis of HCC patients [5]. Overexpression of lncRNA-HOXD-AS1 could competitively bind to miR-130a-3p, thereby preventing the miRNA-mediated degradation of SOX4, up-regulating the expression of EZH2 and MMP2, and promoting HCC metastasis.

Long intergenic non-protein coding RNA 852 (LINC00852) could target on S100A9 and promote the progression and carcinogenicity of lung adenocarcinoma and spinal metastasis (SM) through activating the MAPK pathway [6]. LINC00852 is the most differentially expressed lncRNA in receptor tyrosine kinase AXL-related exosomes, which can partially up-regulate the AXL expression by competitively binding with miR-7-5p, and AXL-related exosomes LINC00852 up-regulate the proliferation, migration and invasion of osteosarcoma cells [7]. It has been reported that LINC00852 is highly expressed in prostate cancer tissues, and lentivirus-mediated overexpression of LINC00852 significantly improves the proliferation, migration and invasion capabilities of the prostate cancer cells [8]. However, the regulatory mechanism of LINC00852 is still unclear.

Based on the target prediction using bioinformatics analysis, LINC00852 was predicted as ceRNA of miR-29a-3p, and the miR-29a-3p has been proved as a tumor suppressor in a variety of cancers from previous studies. Su et al. proved that miR-29a-3p inhibited the proliferation of laryngeal cancer cells by targeting PROM 1 [9]. miR-29a-3p also inhibited the growth, proliferation and invasion of papillary thyroid carcinoma by directly targeting OTUB2, and inhibiting the NF-κB signaling pathway [10]. In prostate cancer, miR-29a-3p could down-regulate the SLC25A15 expression and inhibit the progression, migration and invasion of prostate cancer cells [11, 12]. However, the mechanism of action between miR-29a-3p and LINC00852 in prostate cancer progression is still unclear.

In this study, we probed the mechanism of LINC00852 in promoting the proliferation, migration and invasion of prostate cancer cells, which provided a theoretical basis of LINC00852 as a drug target for prostate cancer treatment.

## Materials and methods

### Sample collection

All of the experiments were approved by the Institutional Review Board and Ethics Committee of the First Hospital of China Medical University. And all patients signed an informed consent form. Sixty pairs of prostate cancer tissues and corresponding adjacent tissues were collected and stored at − 80 °C. None of the patients had received preoperative chemotherapy or radiotherapy.

### Cell culture and treatment

The prostate cancer cell lines LNCaP, VCaP, PC-3 and normal prostate epithelial cells RWPE-1 were purchased from Cell Bank of the Chinese Academy of Sciences (Shanghai, China). The cells were cultured in 10% fetal bovine serum, 100 U/mL penicillin and 100 U/mL streptomycin in DMEM high-sugar medium in an incubator with a constant temperature of 37 °C and 5% CO_2_. The lentiviral vector containing negative control shRNA, LINC00852 shRNA, empty vector and LINC00852 full-length lentiviral vector were designed and synthesized by GenePharma (Shanghai, China).

### Lentiviral transfection

Lentiviral transfection was strictly performed following the instructions. 3 × 10^3^ VCaP cells were inoculated in a 6-well culture plate at 50–60% confluency. Virus and 5 µg/mL Polybrene were added into the complete medium and further cultured for 24 h. Cells were divided into four groups and transfected with different lentiviral vectors, including negative control shRNA (sh-NC), LINC00852 shRNA (sh-LINC00852), empty vector (Lv-NC) and LINC00852 full length (Lv-LINC00852).

### Quantitative real-time PCR (qRT-PCR)

Total RNA was extracted using RNAiso Plus (9108, Takara, Japan). The mRNA was reversely transcribed into complementary DNA with PrimeScript RT Master Mix (RR036A, Takara, Japan). The expression levels of LINC00852, miR-29a-3p and JARID2 mRNA were detected via TB Green® Premix Ex Taq™ II (RR820Q, Takara, Japan). GAPDH or U6 were applied as internal controls. The relative expression levels of genes were calculated with the 2^−ΔΔCt^ method. The primers were shown in Table 1.

**Table 1:** Primers for quantitative real-time PCR

Gene name	Primer sequences
LINC00852	Forward: 5′-CGTTGCCTACAGTCAAGTCAGT-3′ Reverse: 5′-GCCATGGTTCCCTTACTGATAC-3′
miR-29a-3p	Forward: 5′-TGCGGACTGATTTCTTTTGG-3′ Reverse: 5′-CCAGTGCAGGGTCCGAGGT-3′
JARID2	Forward: 5′-GCTTCCCACCAGGATGACAG-3′ Reverse: 5′-TAGCTGGAGGGGGTAGCAAT-3′
GAPDH	Forward: 5′-CGGAGTCAACGGATTTGGTCGTAT-3′ Reverse: 5′- AGCCTTCTCCATGGTGGTGAAGAC-3′
U6	Forward: 5′-TGCGGGTGCTCGCTTCGGCAGC-3′ Reverse: 5′-CCAGTGCAGGGTCCGAGGT-3′

### Western blot assay

The proteins in cultivated cells were extracted by RIPA Lysis Buffer (P0013B, Beyotime, China), and the concentrations of which were measured by BCA Protein Assay Kit (P0010, Beyotime, China). Protein extracts were reserved over SDS-polyacrylamide gel and transferred to PVDF membranes. Primary antibodies anti-JARID2 (1:1000, MA5-38546, Invitrogen, USA), anti-E-cadherin (1:1000, 3195, CST, USA), anti-N-cadherin (1:1000, 13,116, CST, USA), anti-MMP-2 (1:1000, 40,994, CST, USA), anti-MMP-9 (1:1000, 13,667, CST, USA) and anti-GAPDH (1:2000, MA1-16757, Invitrogen, USA) were used to incubate membranes overnight at 4 °C. HRP-conjugated Goat Anti-Rabbit secondary antibodies were incubated with the membranes for 1 h at room temperature. The signals were visualized using BeyoECL Plus (P0018S, Beyotime, China) and a gel imaging system, and Image J was used to calculate the gray values ​​of the images.

### Cell counting kit-8 (CCK-8) assay

CCK-8 assay was used to measure the cell proliferation. Cells in each group were seeded in 96-well plates at a concentration of 2 × 10^3^ cells per well, and left to adhere for 4 h. Then cells were incubated for 24 h, 48 or 72 h. At 0, 24, 48 and 72 h, the culture medium was discarded, and CCK-8 detection reagent was added. After incubated at 37 °C for 1 h, the absorbance of cells was measured at 450 nm using a microplate reader.

### Colony formation assay

The cells of each group were inoculated into a 3.5 cm petri dish at a density of 1000 cells/dish. After cultured at 37 °C, 5% CO_2_ for 2 weeks, the cells were washed using PBS, and fixed with 4% paraformaldehyde for 15 min. Then the cells were stained with 0.1% crystal violet for 30 min, and observed using optical microscope, or counted using Image J software. The percentage rate of colony formation = number of colonies/number of seeded cells *100%.

### Cell invasion and migration assays

For transwell migration assays, cells were transferred to the top chamber of a noncoated membrane chamber in DMEM medium containing 5% fetal calf serum. And for transwell invasion assays, cells were transferred to the top chamber of a Matrigel-coated invasion chamber in DMEM medium containing 5% fetal calf serum. DMEM containing 20% ​​fetal calf serum was added to the lower chamber to act as a chemoattractant. After incubated for 24 h, noninvasive cells were removed from the upper well, and the remaining invasive cells were fixed with 4% paraformaldehyde and stained with crystal violet. The cells were observed and photographed using an optical microscope.

### Dual-luciferase reporter gene assay

Luciferase gene plasmids were purchased from GenePharma (Shanghai, China). The cells were plated at a density of about 50%. Then the cells were transfected with the firefly luciferase plasmid and Renilla luciferase plasmid. Twenty-four hours after transfection, Dual-Glo Luciferase Assay System (E2920, Promega, USA) and GloMax® 20 /20 Luminometer (E5311, Promega, USA) were used to detect the firefly and Renilla luciferase activity.

### Animal studies

Twenty, six-week-old female nude mice were purchased from Beijing Biocytogen Co.,Ltd (Beijing, China). The procedures for the handling and care of the mice were approved by the Animal Experimentation Ethics Committee of China Medical University. 1 × 10^7^ cell in Matrigel (BD Biosciences, USA) were injected into the right flanks of mice to form xenograft tumors. The average volume of the tumor was measured three times in each 3 days. Tumor volumes were calculated using the formula: (L x W^2^)/2.

### Immunohistochemistry staining

After the mice were euthanized by cervical dislocation, tumors were excised from the mice and fixed in 4% paraformaldehyde, then embedded in paraffin. Tissue specimens were successively incubated with antibodies against Ki-67, a biotin-conjugated secondary antibody and an avidin-biotin-peroxidase complex. Visualization was performed using amino-ethyl carbazole chromogen. Slides were analyzed using the Olympus BX43 microscope system (Olympus, Japan).

### Statistical analysis

Results were expressed as mean ± standard deviation (SD) of three independent experiments unless otherwise specified. Data were analyzed using Graph Prism 8.2 software (GraphPad Prism, USA). Student’s t-test was used to analyze differences between groups. One-way analysis of variance (ANOVA) with Tukey’s multiple comparison post hoc test was used to compare the data among groups. *p* < 0.05 was considered as significant difference.

## Results

### LINC00852 was highly expressed in prostate cancer

**Fig. 1:**
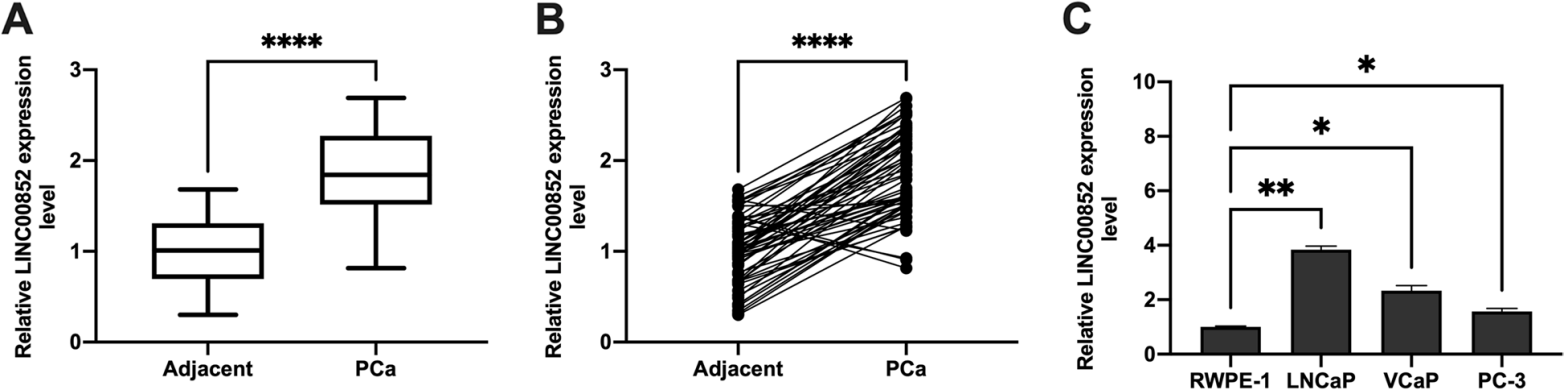
LINC00852 was highly expressed in prostate cancer. Expression of LINC00852 in the (**A**-**B**) prostate cancer and adjacent tissue tissues or (**C**) prostate cancer cell lines and normal prostate epithelial cells that detected using qRT-PCR assay. ****, *p* < 0.0001; **, *p* < 0.01; *, *p* < 0.05

To investigate the function of LINC00852 in prostate cancer, we tested the expression level of LNC00852 in prostate cancer tissues, and cell lines including LNCaP, VCaP, PC-3 and normal prostate epithelial cells (RWPE-1). The qRT-PCR results showed that the expression of LINC00852 was significantly up-regulated in prostate cancer tissues compared with adjacent normal tissues (Fig. 1A-B). And LINC00852 was also highly expressed in prostate cancer cell lines compared to normal prostate epithelial cells RWPE-1 (Fig. 1C).

### LINC00852 promoted the proliferation of prostate cancer cells

**Fig. 2:**
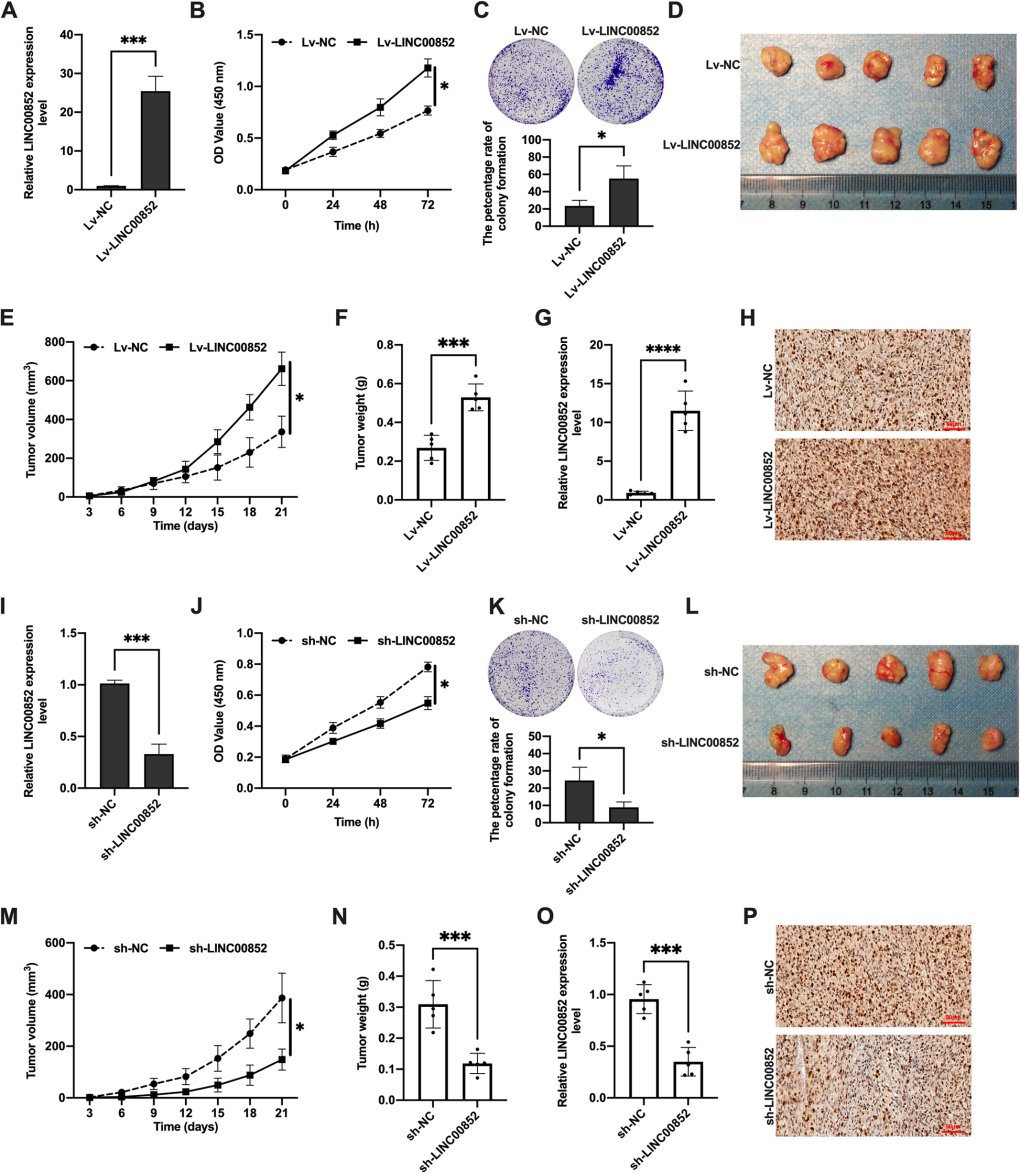
LINC00852 promoted the proliferation of prostate cancer cells in vivo and in vitro. **A** and **I** Expression of LINC00852 in the VCaP cells that detected using qRT-PCR assay. **B** and **J** Proliferation of VCaP cells probed using CCK-8 assay. **C** and **K** Colony formation ability of VCaP cells detected using colony formation test. **D** and **L** Tumors in nude mice. (E and M) Tumor growth curve. **F** and **N** The tumor weight. **G** and **O** Expression of LINC00852 in transplanted tumors detected using qRT-PCR. **H**-**P** Immunohistochemical detection of Ki-67 expression in transplanted tumors. ****, *p* < 0.0001; ***, *p* < 0.001; *, *p* < 0.05

As the level of LINC00852 in VCaP and PC-3 cells was ranged among three prostate cancer cell lines, we selected VCaP and PC-3 cells for the following experiments. In the VCaP (Fig. 2A, I) and PC-3 (Figure S1 A, D) cell line, LINC00852 was efficiently knocked down or overexpressed. The effect of LINC00852 on the proliferation of prostate cancer cells in vitro was detected by CCK-8 and colony formation experiments. CCK-8 results showed that overexpression of LINC00852 increased the proliferation of VCaP (Fig. 2B) and PC-3 (Figure S1 B) cells, and knockdown of LINC00852 decreased the proliferation of VCaP (Fig. 2J) and PC-3 (Figure S1 E) cells. Colony formation experiment further confirmed the above results (Fig. 2C, K and Figure S1 C, F). A nude mouse tumor xenograft model was established to study the effect of LINC00852 on the proliferation of prostate cancer cells in vivo. The results showed that overexpression of LINC00852 promoted the growth of transplanted tumor (Fig. 2D), and the knockdown of LINC00852 had an inhibitory effect on the growth of transplanted tumor (Fig. 2L). Nude mice were sacrificed at 21 days after injection, the transplanted tumor in the Lv-LINC00852 group was significantly larger than that in the Lv-NC group (Fig. 2E), and the weight of tumor was higher than that in the Lv-NC group (Fig. 2F). Interestingly, the transplanted tumor in the sh-LINC00852 group was significantly smaller than the sh-NC group (Fig. 2M), and the tumor weight was lower than the sh-NC group (Fig. 2N). In addition, qRT-PCR results confirmed that the expression of LINC00852 in transplanted tumors in the Lv-LINC00852 group was significantly higher than that in the Lv-NC group (Fig. 2G), and in the sh-LINC00852 group was significantly higher than that in the sh-NC group (Fig. 2O). Immunohistochemical results showed that the proliferation marker Ki-67 was expressed in xenograft tumors in each group. The percentage of Ki-67 positive cells in transplanted tumors in the Lv-LINC00852 group was significantly higher than that in the Lv-NC group (Fig. 2H), while in the sh-LINC00852 group was significantly lower than that in the sh-NC group (Fig. 2P). In summary, the above results indicated that overexpression of LINC00852 promoted the proliferation of prostate cancer cells in vivo and in vitro, while knockdown of LINC00852 inhibited the proliferation of prostate cancer cells in vivo and in vitro.

### LINC00852 promoted the migration and invasion of prostate cancer cells

**Fig. 3:**
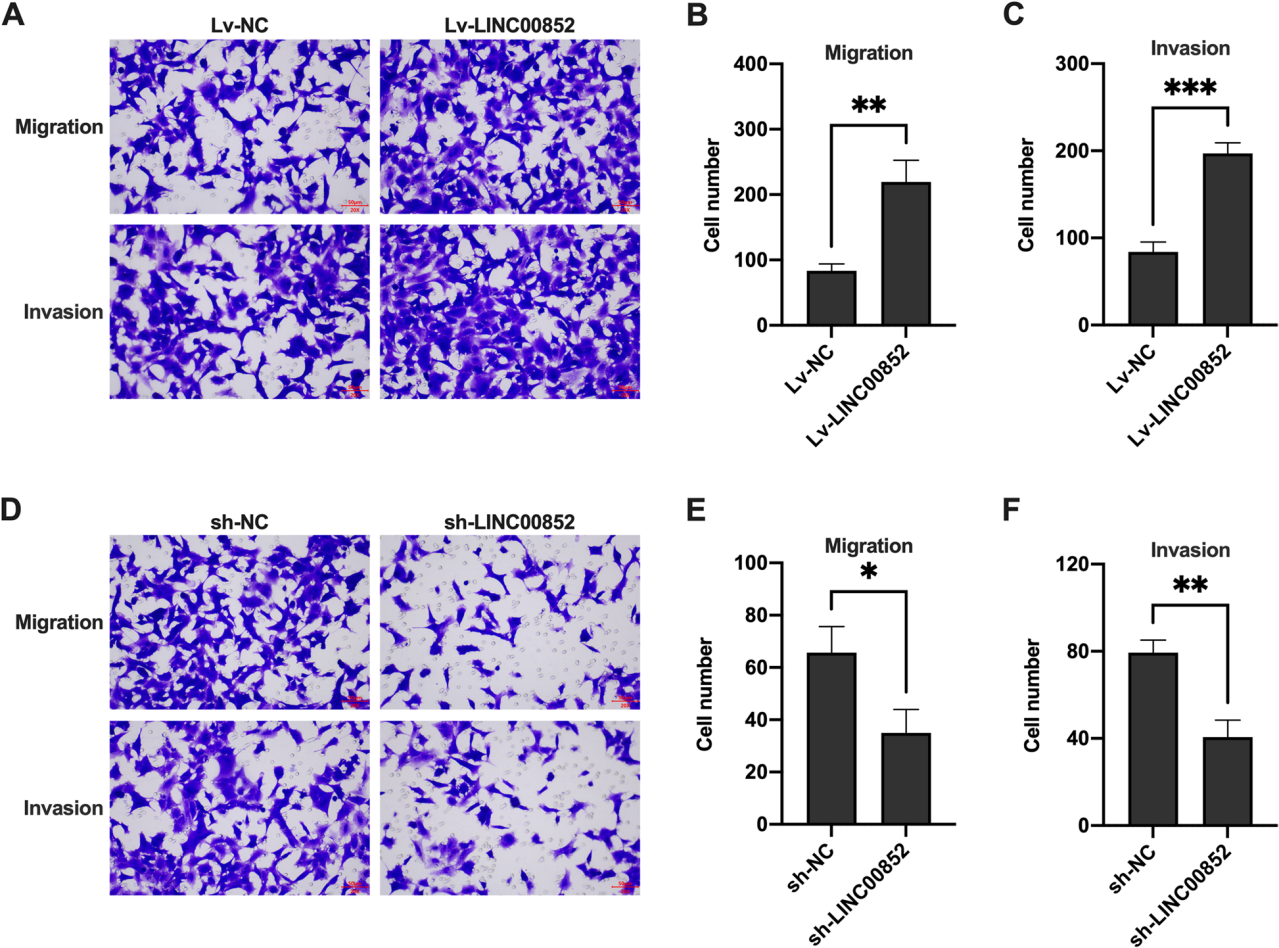
LINC00852 promoted the migration and invasion of prostate cancer cells in vitro. **A** and **D** Migration and invasion of VCaP cells probed using cell transwell migration and invasion assay. **B** and **E** The number of migrated cells. **C** and **F** The number of invasion cells. ***, *p* < 0.001; **, *p* < 0.01; *, *p* < 0.05

The effect of LINC00852 on the migration and invasion of VCaP cells was detected using transwell assay. The results showed that overexpression of LINC00852 significantly increased the migration and invasion of VCaP (Fig. 3A-C) and PC-3 (Figure S2 A-C) cells, and knocking-down of LINC00852 significantly reduced the migration and invasion of VCaP (Fig. 3D-F) and PC-3 cells (Figure S2 D-F). The above results indicated that LINC00852 played a role in the migration and invasion of VCaP and PC-3 cells. Overexpression of LINC00852 significantly promoted the migration and invasion of prostate cancer cells, while knockdown of LINC00852 significantly inhibited the migration and invasion of prostate cancer cells in vitro.

### LINC00852 sponged with miR-29a-3p in prostate cancer cells

**Fig. 4:**
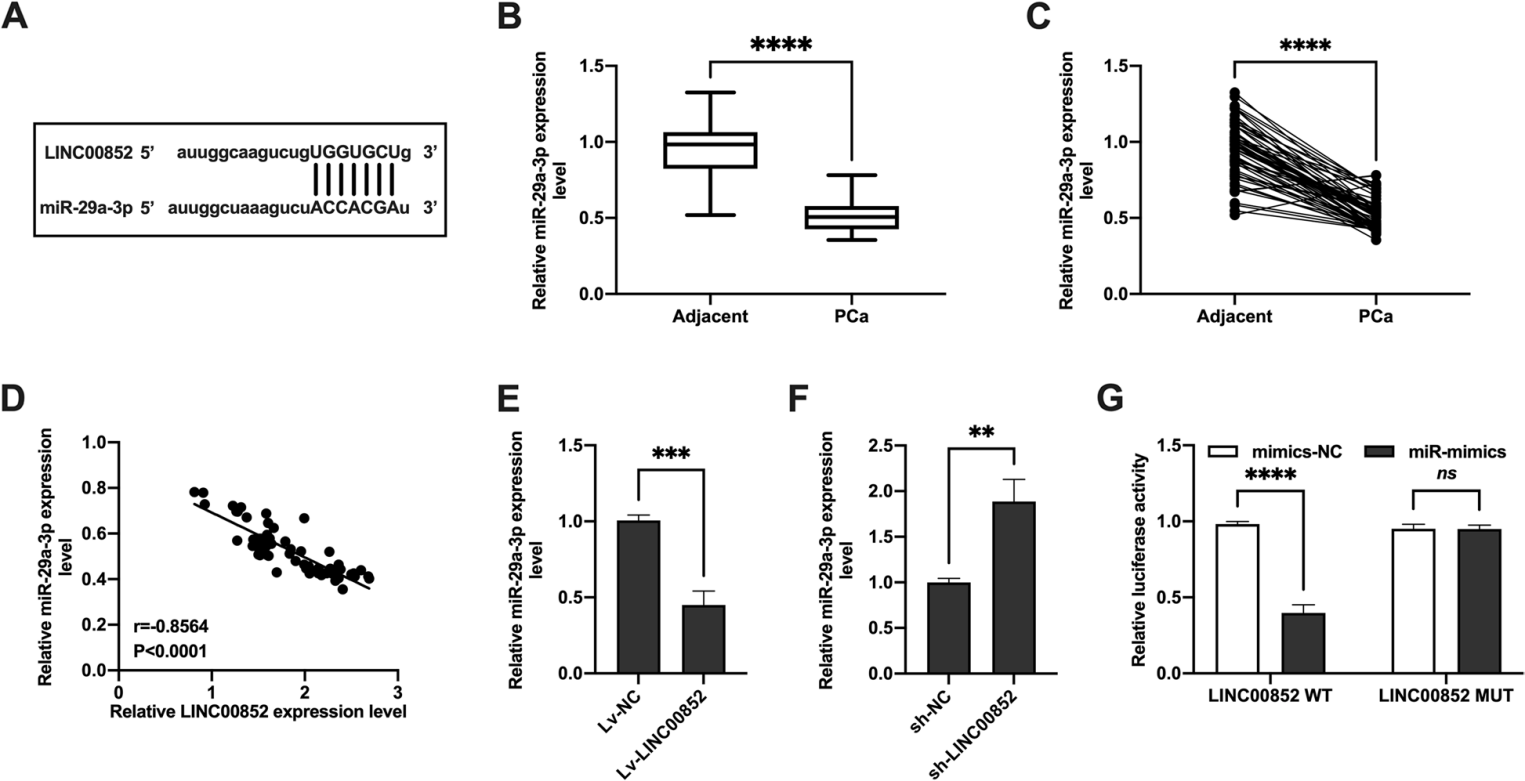
LINC00852 sponged with miR-29a-3p in prostate cancer cells. (A) The potential targeting miRNAs of LINC00852 was analyzed by ENCORI database. (B and C) Expression of miR-29a-3p in prostate cancer and corresponding paracancerous tissues measured using qRT-PCR. (D) Correlation analysis of miR-29a-3p and LINC00852 in prostate cancer tissue. (E and F) Expression of miR-29a-3p in VCaP cells measured using qRT-PCR. (G) Dual-luciferase reporter gene assay. ****, *p* < 0.0001; ***, *p* < 0.001; **, *p* < 0.01; ns, *p* > 0.05

To further study the mechanism of LINC00852 on the proliferation and invasion of prostate cancer cells, the potential target miRNA of LINC00852 was analyzed using ENCORI database (http://starbase.sysu.edu.cn). The results showed that there were potential binding sites between LINC00852 and miR-29a-3p (Fig. 4A). In order to analyze the potential relationship between LINC00852 and miR-29a-3p expression in prostate cancer, we detected the expression of miR-29a-3p in prostate cancer tissue by qRT-PCR. Compared with adjacent tissues, the expression level of miR-29a-3p was significantly down-regulated in prostate cancer tissues (Fig. 4B-C). The results of correlation analysis showed that the expression levels of miR-29a-3p and LINC00852 in prostate cancer tissues were negatively correlated (Fig. 4D). Overexpression of LINC00852 reduced the expression of miR-29a-3p in VCaP cells (Fig. 4E), while knocking down LINC00852 showed opposite effects (Fig. 4 F). The results of dual-luciferase reporter gene assay showed that miR-29a-3p mimics significantly inhibited LINC00852-WT luciferase activity, while showed non-influence on the luciferase activity of LINC00852-MUT (Fig. 4G). In summary, LINC00852 may act as ceRNA and sponge miR-29a-3p in prostate cancer cells.

### LINC00852 regulated the proliferation and invasion of prostate cancer cells through miR-29a-3p

**Fig. 5:**
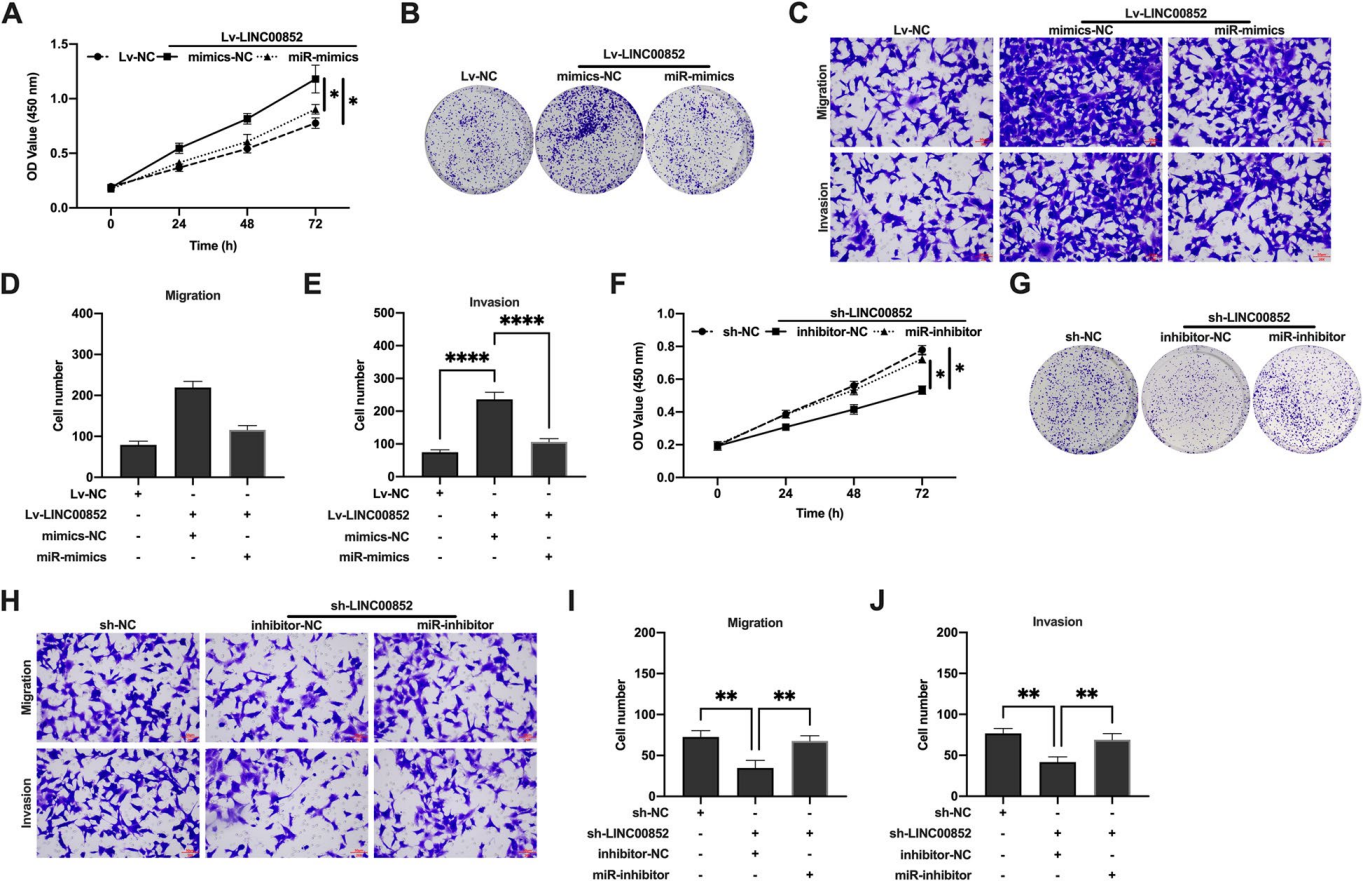
LINC00852 regulated the proliferation and invasion of prostate cancer cells in vitro through targeting miR-29a-3p. **A** and **F** to Proliferation activity of VCaP cells that detected using CCK-8 assay. **B** and **G** Duplication capability of VCaP cells that detected using colony formation assay. **C** and **H** Migration and invasion of VCaP cells probed using transwell migration and invasion assays. **D** and **I** The number of migrated cells. **E** and **J** The number of invaded cells. ****, *p* < 0.0001; **, *p* < 0.01; *, *p* < 0.05

Furthermore, we confirmed whether LINC00852 could regulate the proliferation and invasion of prostate cancer cells through binding miR-29a-3p. The results of CCK-8 and colony formation assays showed that miR-29a-3p mimics inhibited the proliferation of VCaP (Fig. 5A, B) and PC-3 (Figure S3 A, B) cells, while miR-29a-3p inhibitor promoted the proliferation of VCaP (Fig. 5F, G) and PC-3 (Figure S3 F, G) cells. Transwell migration and invasion experiment results showed that miR-29a-3p mimics inhibited the migration and invasion of VCaP (Fig. 5C-E) and PC-3 (Figure S3 C-E) cells, and miR-29a-3p inhibitor promoted the migration and invasion of VCaP (Fig. 5H-J) and PC-3 (Figure S3 H-J) cells. These results indicated that miR-29a-3p might block the effect of LINC00852 on the biological functions of prostate cancer cells in vitro.

### JARID2 was the target protein of miR-29a-3p and indirectly regulated by LINC00852

**Fig. 6:**
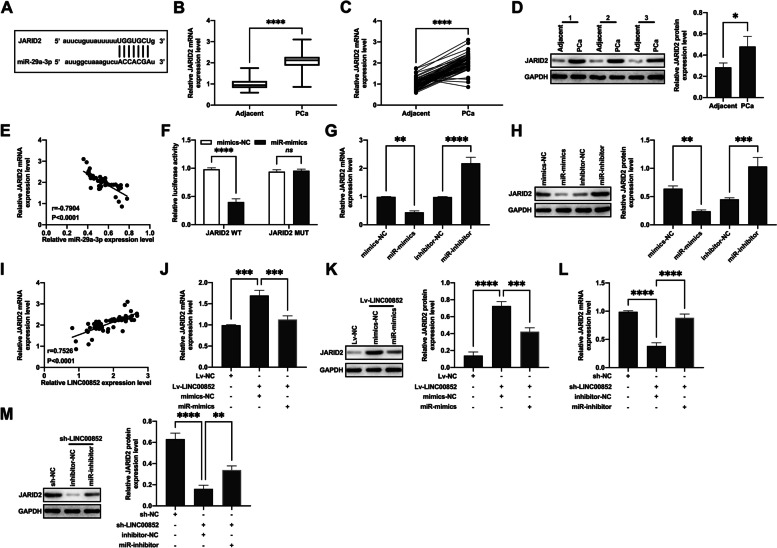
JARID2 was one of target proteins of miR-29a-3p and indirectly regulated by LINC00852. **A** Analysis of the potential target miRNA of LINC00852 through ENCORI database. **B**-**C** mRNA level of JARID2 in prostate cancer and adjacent tissues detected using qRT-PCR. **D** Expression of JARID2 in prostate cancer tissue detected using western blot. **E** Correlation analysis of miR-29a-3p and JARID2 mRNA levels in prostate cancer tissues. **F** Dual-luciferase reporter gene assay results. **G**, **J** and **L** mRNA level of JARID2 in VCaP cells that detected using qRT-PCR. **H**, **K** and **M** Expression of JARID2 in VCaP cells that detected using western blot. **I** Correlation analysis of LINC00852 and JARID2 mRNA levels in prostate cancer tissue. ****, *p* < 0.0001; ***, *p* < 0.001; **, *p* < 0.01; *, *p* < 0.05

From the bioinformatics analysis results (ENCORI), the 3’UTR region of JARID2 mRNA possessed potential binding sites for miR-29a-3p (Fig. 6A). The results of qRT-PCR (Fig. 6B-C) ​​and western blot (Fig. 6D) showed that JARID2 was highly expressed in prostate cancer tissues. And the expression of JARID2 mRNA was negatively correlated with miR-29a-3p (Fig. 6E). The results of dual-luciferase reporter gene assay showed that miR-29a-3p significantly inhibited JARID2-WT luciferase activity, while showed non-influence on the luciferase activity of JARID2-MUT (Fig. 6F). qRT-PCR and Western blot results showed that miR-29a-3p mimics reduced the expression of JARID2 in VCaP (Fig. 6G-H), PC-3 (Figure S4 A-B) and RWPE1 (normal prostate epithelial cells) (Figure S7) cells, and miR-29a-3p inhibitor increased the expression of JARID2. The results of correlation analysis showed that the expression levels of LINC00852 and JARID2 mRNA were positively correlated in prostate cancer tissues (Fig. 6I). Overexpression of LINC00852 increased the expression of JARID2 in VCaP (Fig. 6J, K) and PC-3 (Figure S4 C, D) cells, while knockdown of LINC00852 decreased the expression of JARID2 in VCaP (Fig. 6L, M) and PC-3 (Figure S4 E, F) cells. miR-29a-3p mimics reduced the expression of JARID2 in VCaP (Fig. 6J, K) and PC-3 (Figure S4 C, D) cells, and miR-29a-3p inhibitor increased the expression of JARID2 in VCaP (Fig. 6L, M) and PC-3 (Figure S4 E, F) cells. In conclusion, LINC00852 might regulate JARID2 expression by targeting miR-29a-3p.

### LINC00852 regulated the proliferation and invasion of prostate cancer cells in vitro through miR-29a-3p/JARID2

**Fig. 7:**
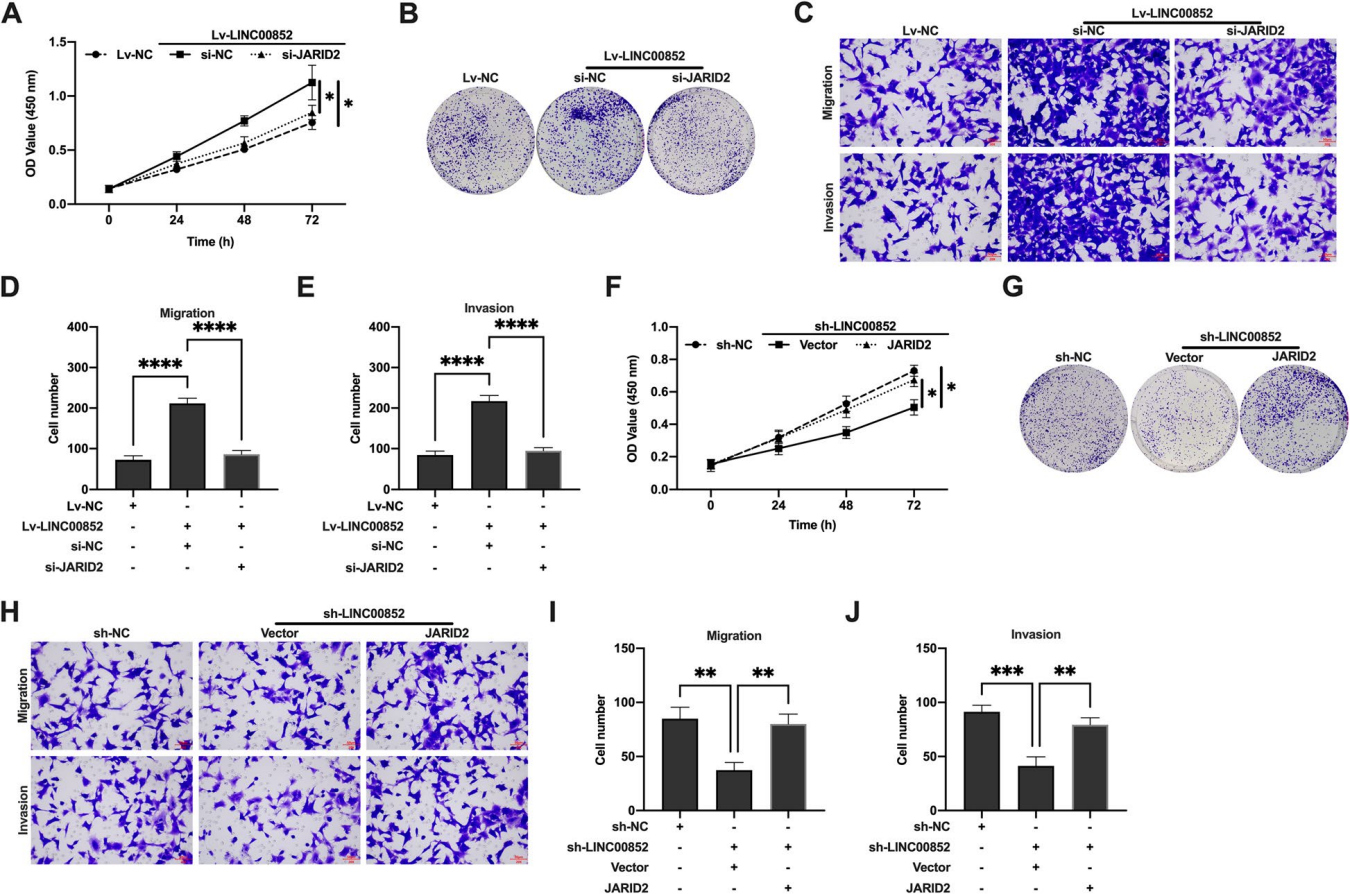
LINC00852 regulated the proliferation and invasion of prostate cancer cells in vitro through regulation of JARID2 expression. **A** and **F** Proliferation activity of VCaP cells probed using CCK-8 assay. **B** and **G** Duplication capability of VCaP cells probed using colony formation assay. **C** and **H** Metastasis capability of VCaP cells probed using transwell migration and invasion assay. **D** and **I** The number of migrated cells. **E** and **J** The number of invaded cells. ****, *p* < 0.0001; ***, *p* < 0.001; **, *p* < 0.01; *, *p* < 0.05

**Fig. 8:**
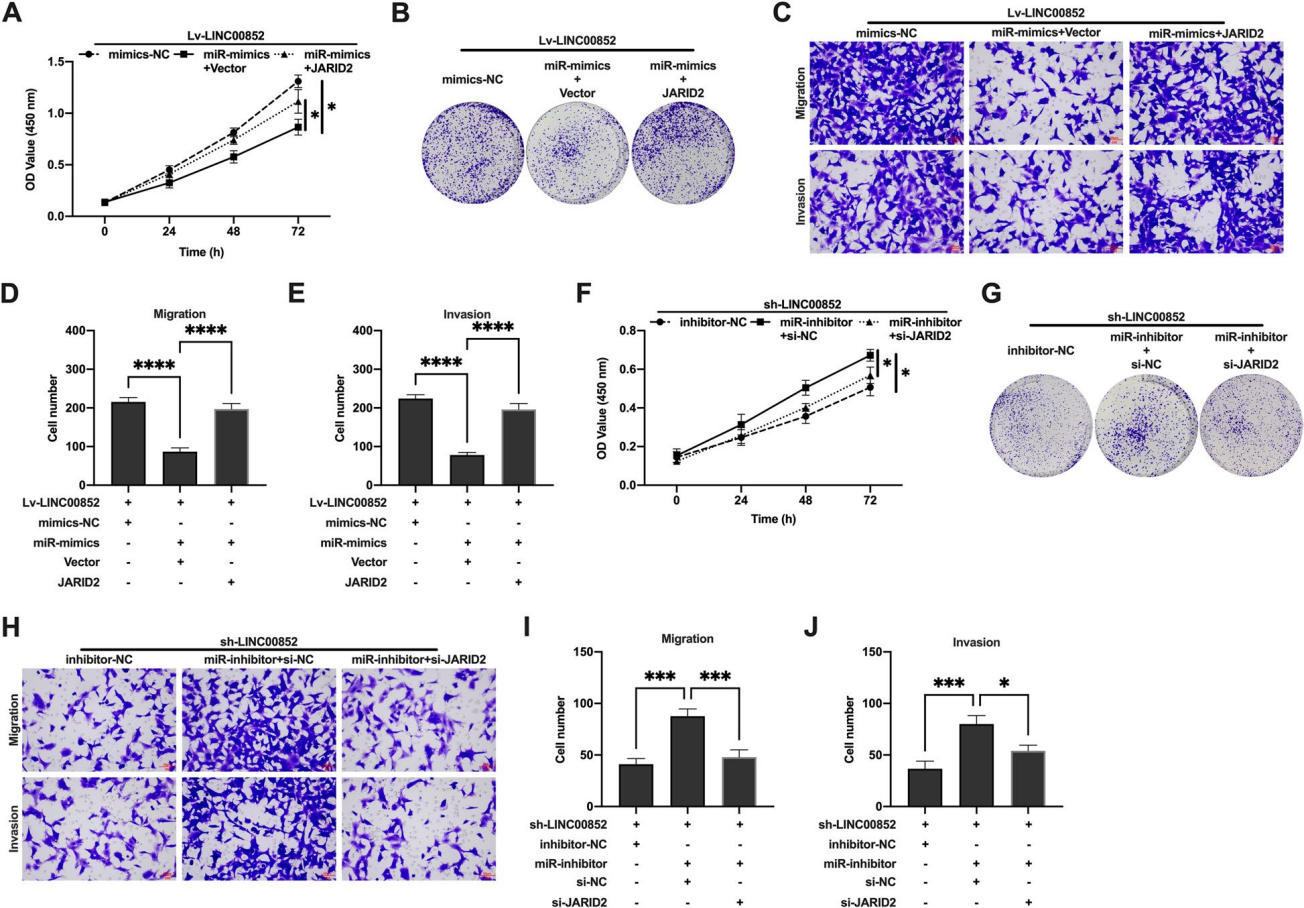
LINC00852 regulated the expression of JARID2 by targeting miR-29a-3p. **A** and **F** Proliferation activity of VCaP cells probed using CCK-8 assay. **B** and **G** Duplication capability of VCaP cells probed using colony formation assay. **C** and **H** Migration and invasion of VCaP cells probed using transwell migration and invasion assay. **D** and **I** The number of migrative cells. **E** and **J** The number of invasive cells. ****, *p* < 0.0001; ***, *p* < 0.001; *, *p* < 0.05

Firstly, we aimed to identify the hypothesis that LINC00852 affects the proliferation and invasion of prostate cancer cells through regulation of JARID2 expression. The CCK-8 and colony formation assays showed that si-JARID2 inhibited the proliferation of VCaP (Fig. 7A, B) and PC-3 (Figure S5 A, B) cells in the Lv-LINC00852 group. And ov-JARID2 promoted the proliferation of VCaP (Fig. 7F, G) and PC-3 (Figure S5 D, E) cells in the sh-LINC00852 group. Transwell migration and invasion experiments showed that si-JARID2 inhibited the migration and invasion of VCaP (Fig. 7C-E) and PC-3 (Figure S5 C) cells in the Lv-LINC00852 group. On the contrary, ov-JARID2 promoted the migration and invasion of VCaP (Fig. 7H-J) and PC-3 (Figure S5 F) cells in the sh-LINC00852 group. Furthermore, we analyzed whether LINC00852 regulated the expression of JARID2 by targeting miR-29a-3p. The experimental results showed that overexpression of JARID2 reversed the inhibitory effects of miR-29a-3p mimics on the proliferation, invasion and migration of VCaP (Fig. 8A-E) and PC-3 (Figure S6 A-C) cells in the Lv-LINC00852 group. And silencing JARID2 partially blocked the promotion effect of VCaP (Fig. 8F-J) and PC-3 (Figure S6 D-F) cells that induced by miR-29a-3p inhibitor on sh-LINC00852 group. In addition, in vivo experiments showed that knockdown of LINC00852 increased xenograft tissues miR-29a-3p expression and decreased JARID2 mRNA expression in VCaP cells. Conversely, overexpression of LINC00852 decreased xenograft tissues miR-29a-3p expression in VCaP cells and increased JARID2 mRNA expression (Figure S8). In summary, overexpression of LINC00852 might promote the proliferation, invasion and migration of prostate cancer cells through miR-29a-3p/JARID2 axis, and the knock-down of LINC00852 could reverse this phenotype.

## Discussion

Prostate cancer is one of the leading causes of morbidity and death among men worldwide [13], the morbidity and mortality of which are among the top five cancers in the world [14]. Proliferation, migration and invasion are the three major characteristics of malignant tumor cells, and metastatic disease is the main cause of prostate cancer-related death [2]. The consequences of prostate cancer metastasis remain severe, with a huge impact on on patient mortality and overall quality of life [15]. Poor prognosis for patients due to the spread of tumor. It is estimated that 98% of patients suffered from prostate cancer metastasis have an overall survival of less than 5 years [16]. Distant metastases from prostate cancer occur primarily in the skeleton, and ara osteolytic, sclerotic (osteoblastic) or mixed osteolytic/sclerotic [17]. Other organs that have metastasized include lymph nodes, liver, lungs and brain [18–20]. Our research showed that LINC00852 was highly expressed in prostate cancer tissues and cell lines. Furthermore, we confirmed that overexpression of LINC00852 promoted the proliferation, migration and invasion of prostate cancer cells, and knocking-down of LINC00852 could inhibited the progression of prostate cancer cell, suggesting that LINC00852 played a cancer-promoting effect in prostate cancer, these results were consistent with the report of Yi et al. [8]. In this study, we further analyzed the mechanism of LINC00852 in promoting the proliferation, migration and invasion of prostate cancer cells.

LncRNA can act as a sponge of miRNA and regulate the activity of miRNA on its target mRNA. LncRNA competes with mRNA for binding to the target miRNA, thereby reducing the inhibitory effect of miRNA on mRNA [21]. Yu et al. reported that lncRNA UCA1, as a ceRNA, promoted the enhancement of prostate cancer progression though sponging miR-143 in the PCa study [22]. LncRNA KCNQ1OT1 was also proved to promote cell progression of prostate cancer through targeting on miR-211-5p/CHI3L1 axis [23]. In addition, lncRNA HAND2-AS1 could inhibit PCa Cell proliferation by regulating miR-106a-5p/RBM24 signaling pathway [24]. In this study, we confirmed the binding of LINC00852 with miR-29a-3p using dual-luciferase reporter gene assay, and LINC00852 negatively regulated miR-29a-3p in prostate cancer cells. Previous studies have shown that miR-29a-3p inhibited the proliferation, migration and invasion of a variety of cancer cells [25–27]. For example, miR-29a-3p was reported to target on DVL3 and inhibit the proliferation, migration and invasion of colorectal cancer cells [25]. miR-29a-3p also inhibited the epithelial-mesenchymal transition in ovarian cancer through targeting on the mRNA of COL1A1 [26]. miR-29a-3p/GUCD1 axis inhibited the proliferation, migration and invasion of liver cancer cells [27]. Therefore, the regulation of LINC00852 on miR-29a-3p may be a potential strategy to inhibit PTC metastasis. Transfection of miR-29a-3p mimics reversed the effect of overexpression of LINC00852 on the proliferation, migration and invasion of prostate cancer cells to further confirm this result.

To further investigate the effects of LINC00852 and miR-29a-3p on prostate cancer, we studied the downstream regulatory mechanism of miR-29a-3p in the progression of prostate cancer. The bioinformatics analysis showed that there were potential binding sites for miR-29a-3p in the 3’UTR region of JARID2 mRNA. The dual-luciferase reporter gene assay was used to identify the binding of 3’UTR of JARID2 mRNA and miR-29a-3p. Meanwhile, transfection of miR-29a-3p mimics could inhibit the expression of JARID2. It was indicated that JARID2 was the target gene of miR-29a-3p. The JARID2 protein belonged to Jumonji family, and was the most widely studied PRC2 accessory protein. Studies have shown that JARID2 was highly expressed in a variety of cancers, and played a role in promoting cancer. Wang et al. showed that JARID2 was highly expressed in bladder cancer tissues and cells, and knocking down of which could significantly inhibit the proliferation, migration and tumorigenesis of bladder cancer cells, and resulting cell apoptosis [28]. JARID2 was also reported to be highly expressed in human ovarian cancer cell lines, and downregulation of JARID2 could significantly inhibit the cell proliferation, migration, invasion and epithelial-mesenchymal transition [29]. In this study, we demonstrated that the expression of JARID2 was indirectly regulated by LINC00852. Overexpression of LINC00852 could increase the expression of JARID2 in prostate cancer cells, and miR-29a-3p mimics reversed the cell promotion under overexpression of LINC00852 through targeting on JARID2 in prostate cancer cells. Moreover, silencing JARID2 reversed the proliferation, migration and invasion of prostate cancer cells by overexpression of LINC00852.

Our studies were partially in line with the results of Yi et al., in which, they found LINC00852 was highly expressed in prostate cancer in TCGA database, but did not verify it at prostate cancer patients’ tissue. In addition, overexpression of LINC00852 was only proved to regulate the biologic processes in prostate cancer cell, while the detailed mechanism was not fully demonstrated [8]. In our study, LINC00852 was indicated that with the ability to promote the progression of prostate cancer and exert a malignant effect as a ceRNA through the miR-29a-3p/JARID2 axis. Collectively, our results indicated that the LINC00852/miR-29a-3p/JARID2 axis might play a key role in the progression of prostate cancer. Thus, LINC00852 is a promising target for the treatment of prostate cancer.

## Supplementary Information

**Additional file 1:** **Figure S1.** LINC00852 promotes the proliferation of PC-3 cells in vitro.

**Additional file 2:** **Figure S2.** LINC00852 promotes the migration and invasion of PC-3 cells in vitro.

**Additional file 3:** **Figure S3.** LINC00852 regulates the proliferation and invasion of PC-3 cells in vitro through targeting miR-29a-3p.

**Additional file 4:** **Figure S4.** JARID2 indirectly regulated by LINC00852/miR-29a-3p in PC-3 cells.

**Additional file 5:** **Figure S5.** LINC00852 regulates the proliferation and invasion of PC-3 cells in vitro through regulation of JARID2 expression.

**Additional file 6:** **Figure S6.** LINC00852 regulates the expression of JARID2 by targeting miR-29a-3p in PC-3 cells.

**Additional file 7:** **Figure S7.** JARID2 regulated by miR-29a-3p in normal prostate epithelial cells RWPE1.

**Additional file 8:** **Figure S8.** Knockdown of LINC00852 increased xenograft tissues miR-29a-3p expression and decreased JARID2 mRNA expression in VCaP cells.

**Additional file 9:** Original bands for Western blot.

## Data Availability

The datasets used and/or analyzed during the current study are available from the corresponding author on reasonable request.

## Declarations

The animal experiments reported in the manuscript were approved by the Animal Experimentation Ethics Committee of China Medical University, China‬. All experiments were performed in accordance with relevant guidelines and regulations. All methods are reported in accordance with ARRIVE guidelines (https://arriveguidelines.org) for the reporting of animal experiments.‬‬‬‬.
