# Supplementary figures and images for: The LINC00852/miR-29a-3p/JARID2 axis regulates the proliferation and invasion of prostate cancer cell
Source: BMC Cancer. 2022 Dec 5;22:1269. doi: 10.1186/s12885-022-10263-6 (PMC9724404; doi:10.1186/s12885-022-10263-6)

**A**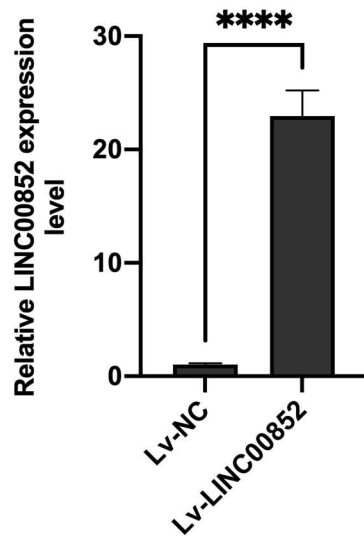**B**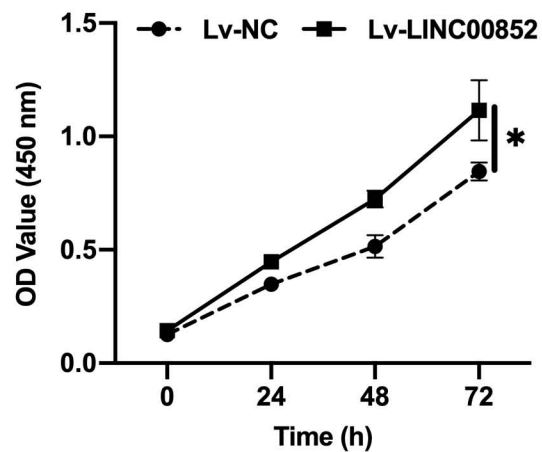**C**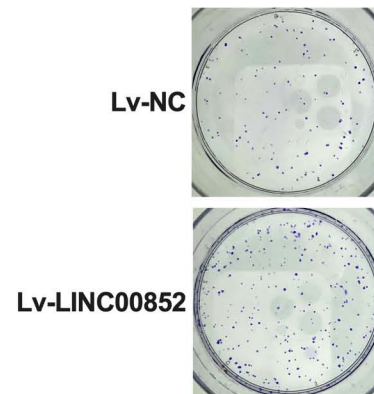**D**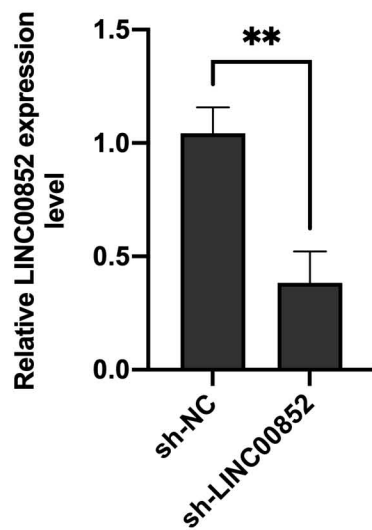**E**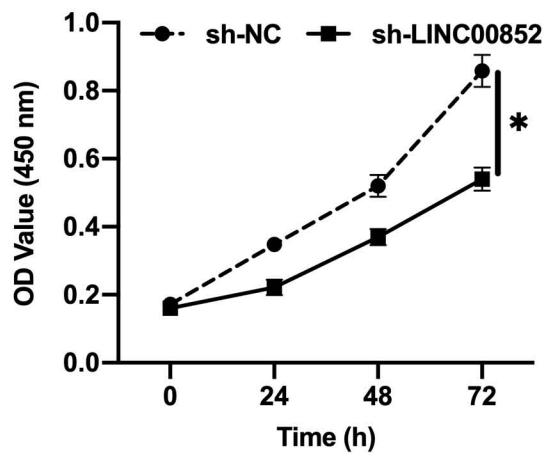**F**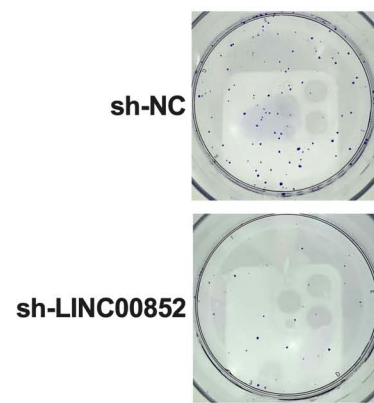

Supplement: Supplementary file 1 — Additional file 1: Figure S1. LINC00852 promotes the proliferation of PC-3 cells in vitro. [file 12885_2022_10263_MOESM1_ESM.pdf]

**A****Lv-NC****Lv-LINC00852****Migration**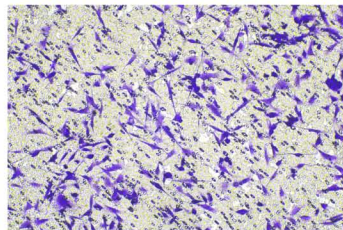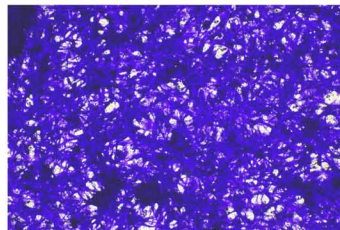**Invasion**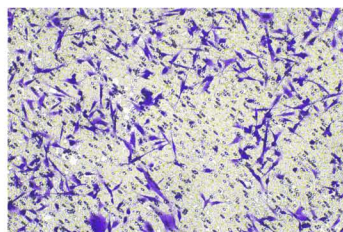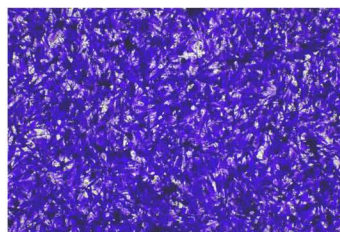**B**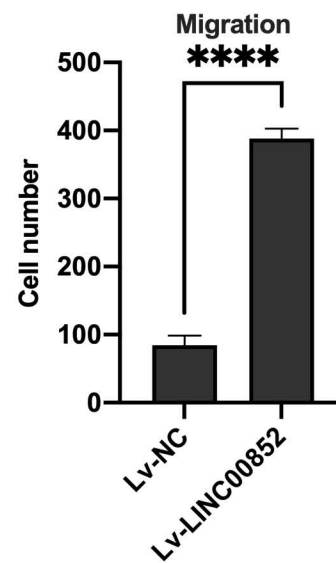**C**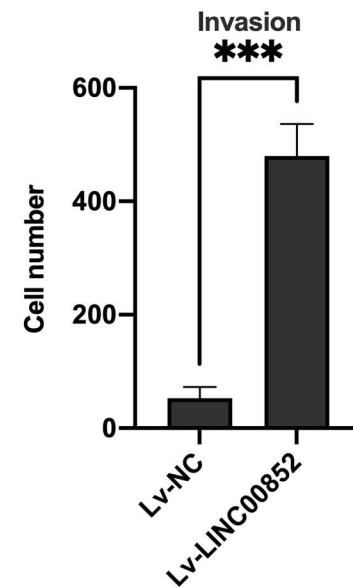**D****sh-NC****sh-LINC00852****Migration**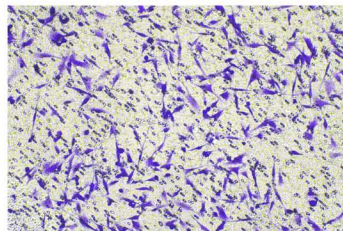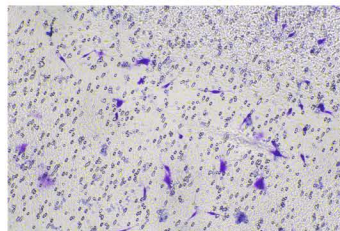**Invasion**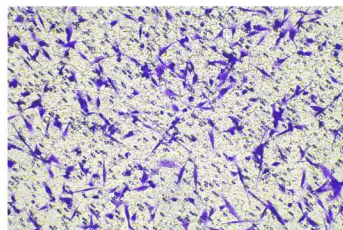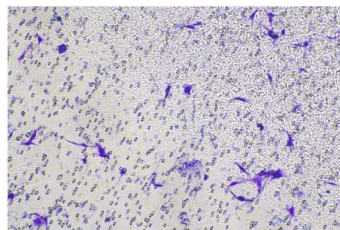**E**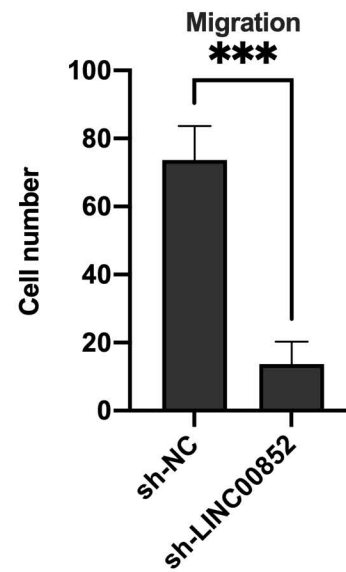**F**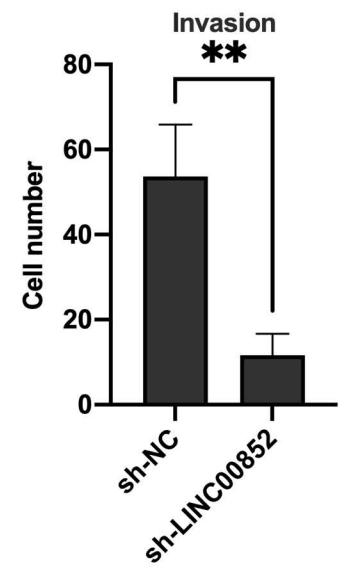

Supplement: Supplementary file 2 — Additional file 2: Figure S2. LINC00852 promotes the migration and invasion of PC-3 cells in vitro. [file 12885_2022_10263_MOESM2_ESM.pdf]

A

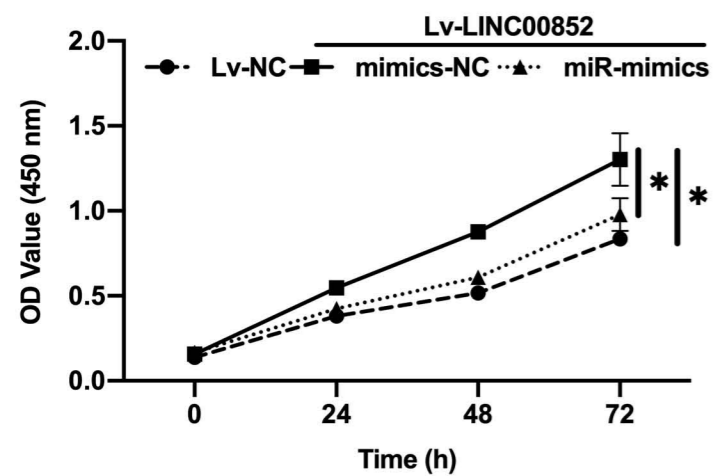

B

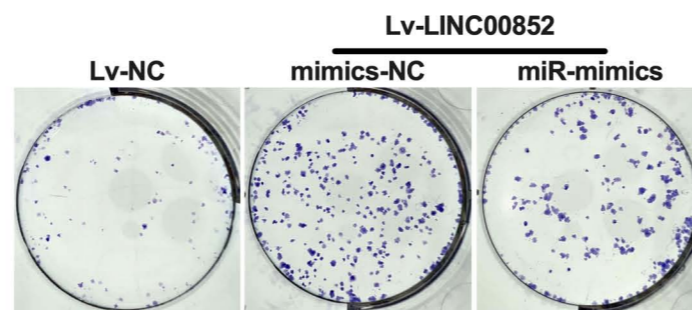

C

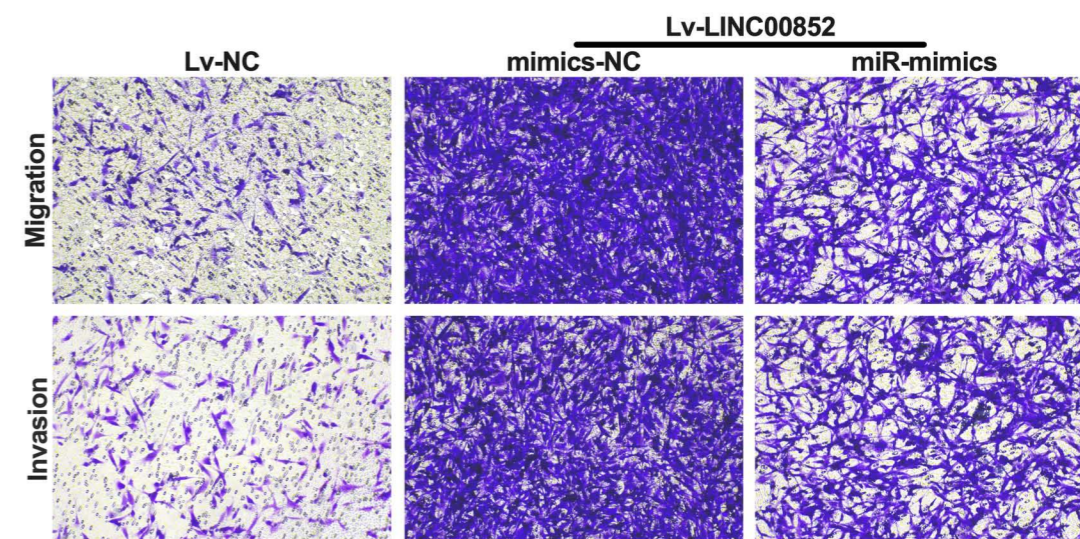

D

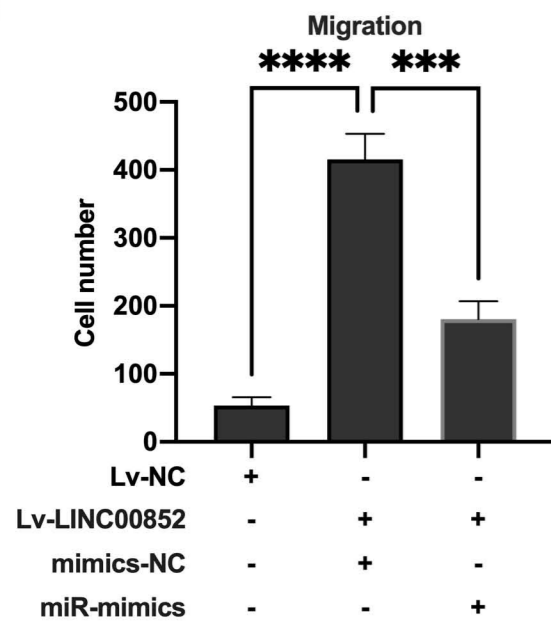

E

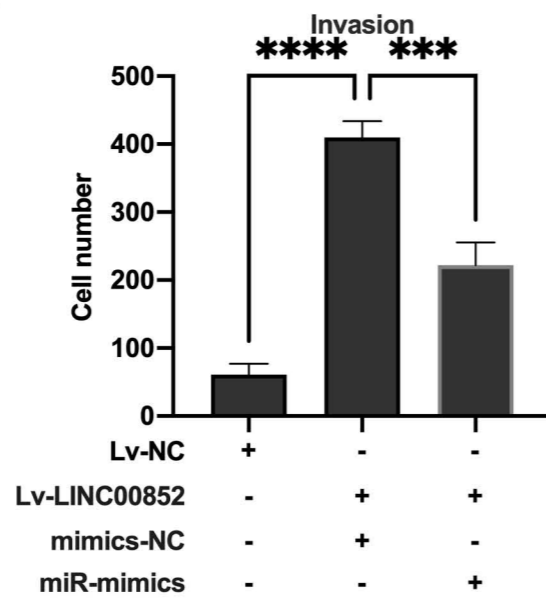

F

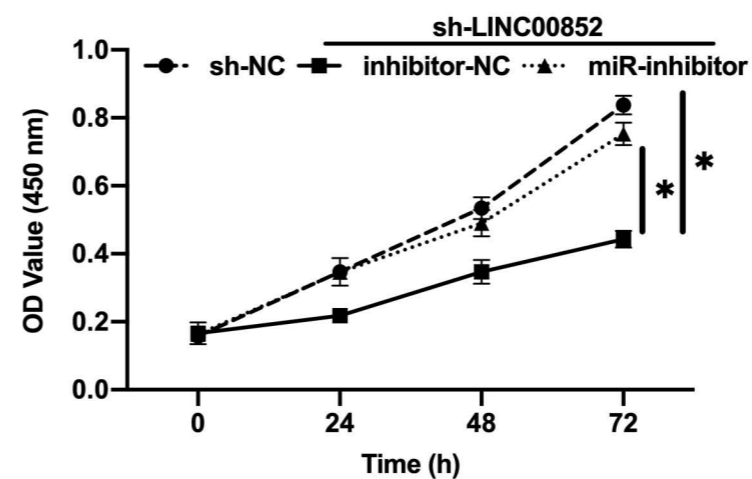

G

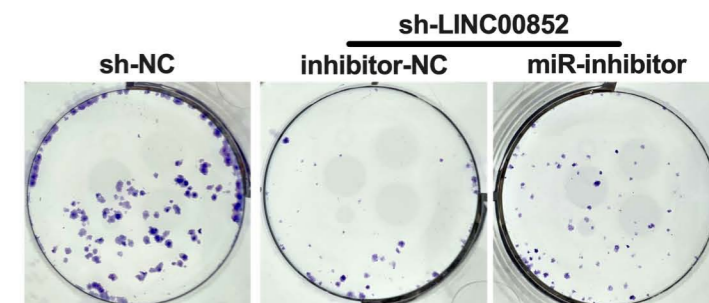

H

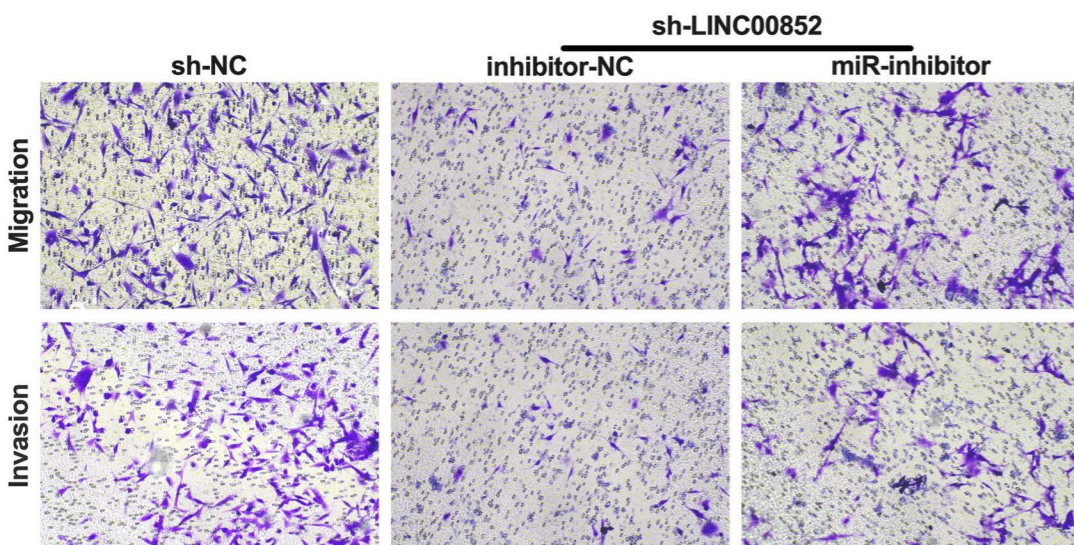

I

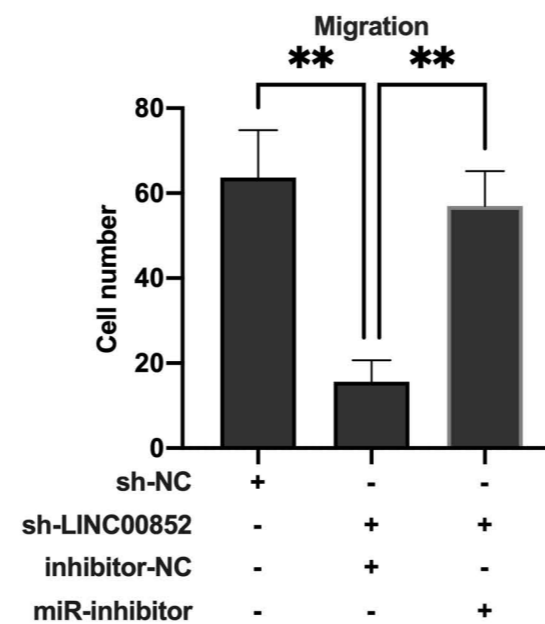

J

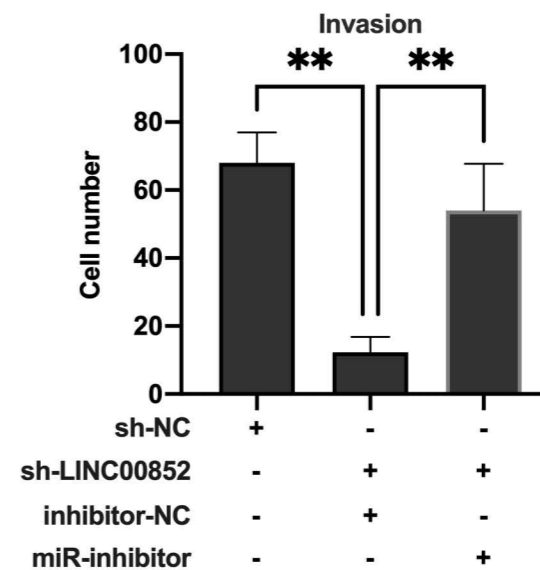

Supplement: Supplementary file 3 — Additional file 3: Figure S3. LINC00852 regulates the proliferation and invasion of PC-3 cells in vitro through targeting miR-29a-3p. [file 12885_2022_10263_MOESM3_ESM.pdf]

A

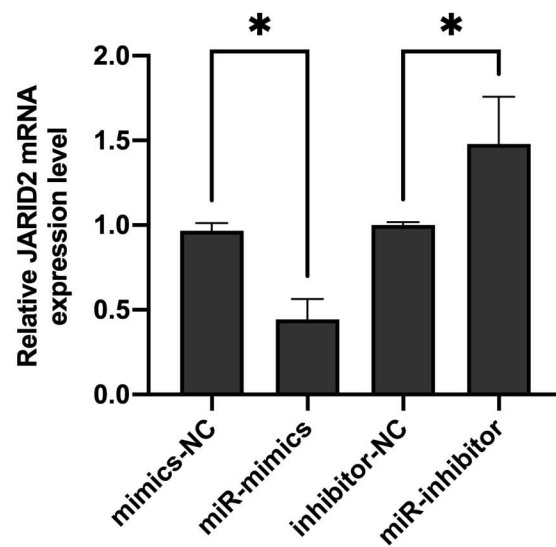

B

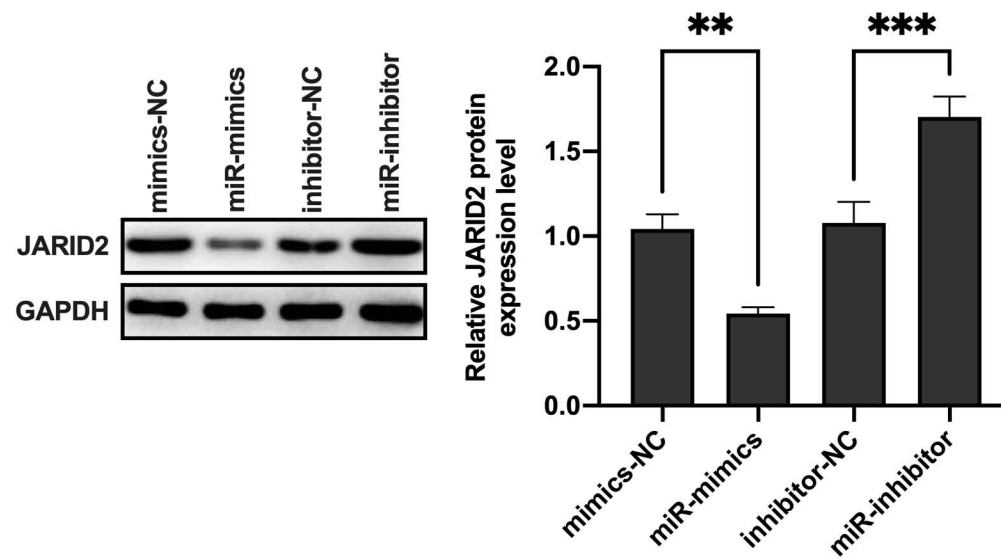

C

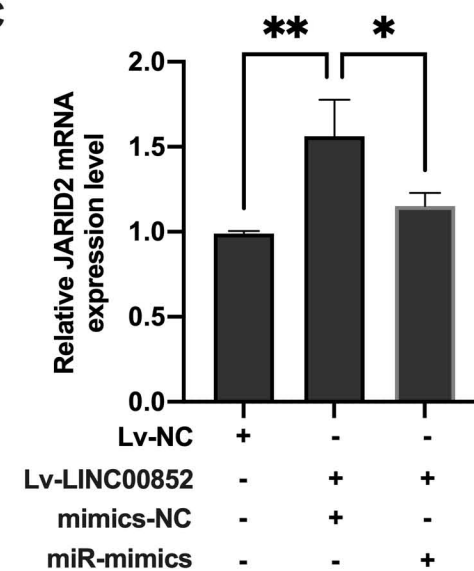

D

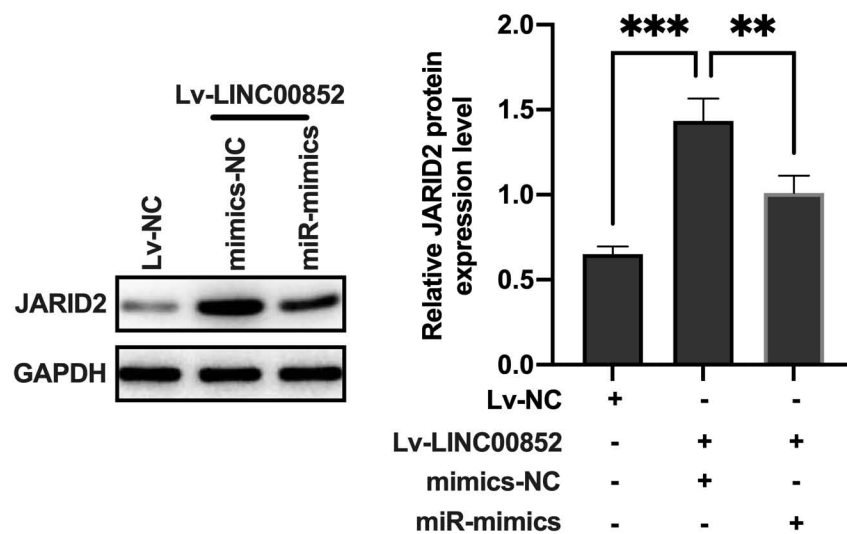

E

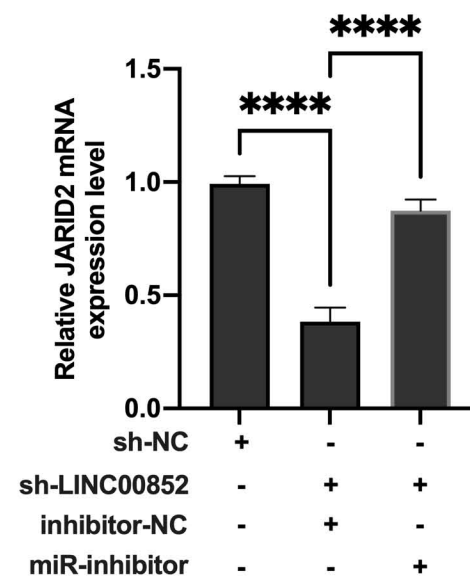

F

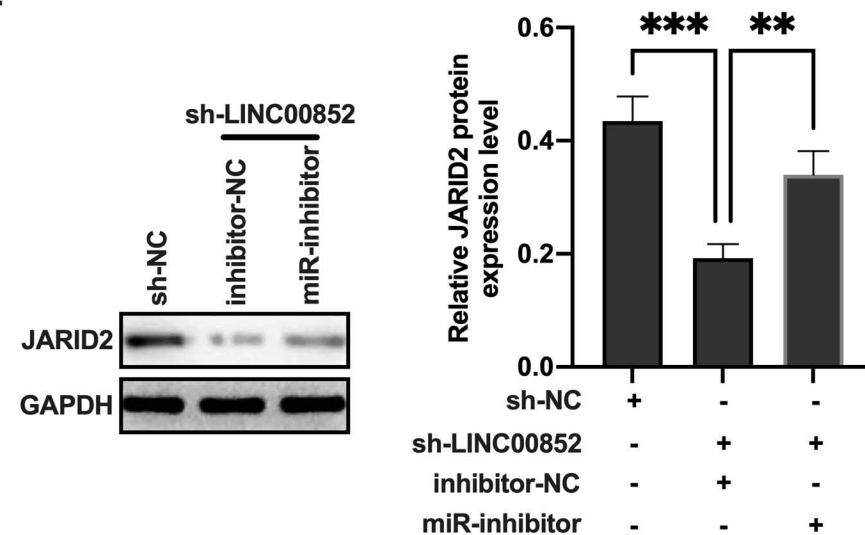

Supplement: Supplementary file 4 — Additional file 4: Figure S4. JARID2 indirectly regulated by LINC00852/miR-29a-3p in PC-3 cells. [file 12885_2022_10263_MOESM4_ESM.pdf]

**A**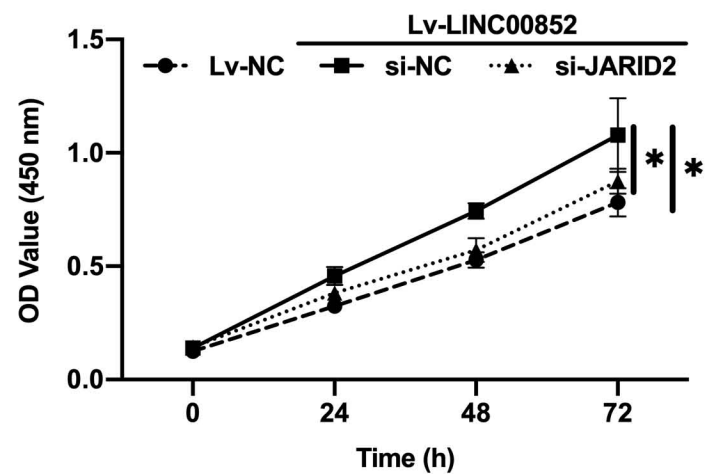**B**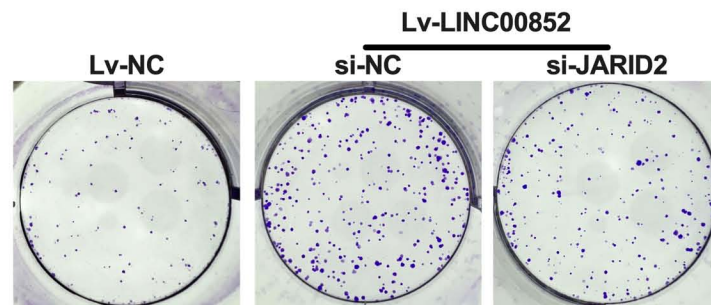**C**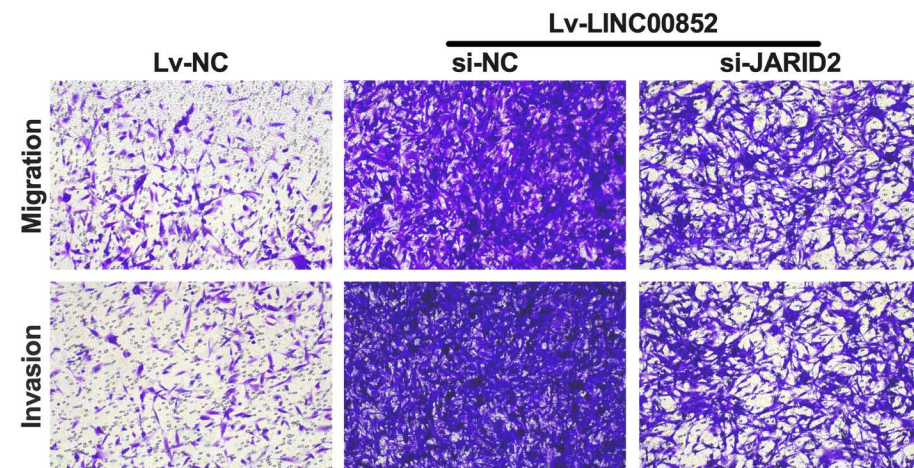**D**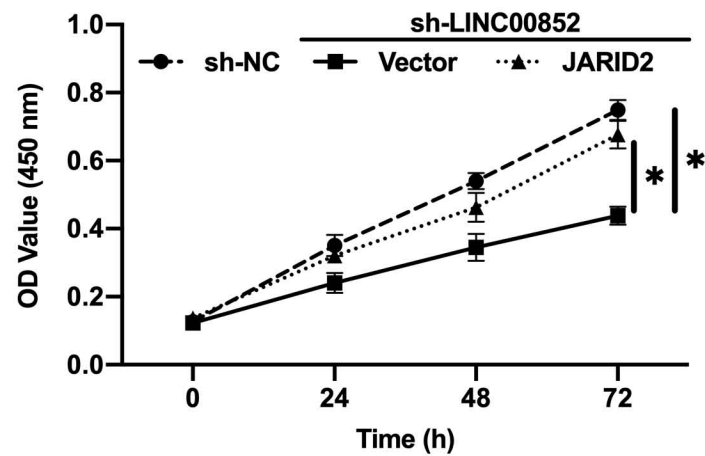**E**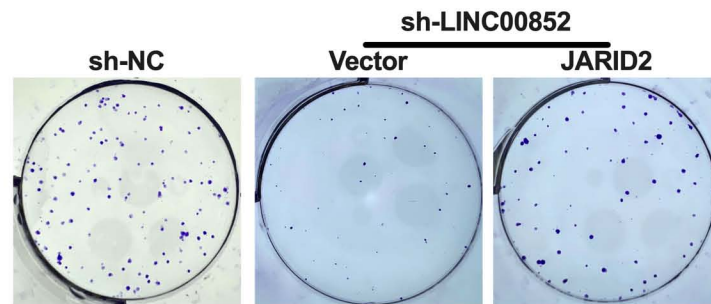**F**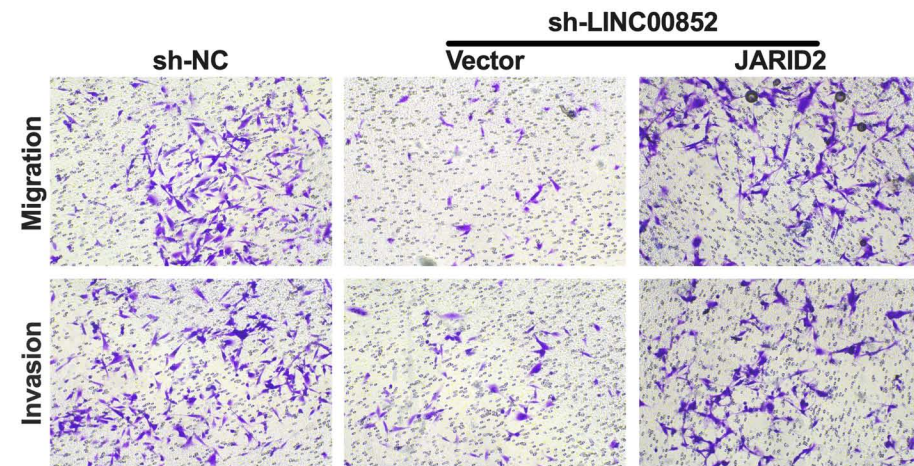

Supplement: Supplementary file 5 — Additional file 5: Figure S5. LINC00852 regulates the proliferation and invasion of PC-3 cells in vitro through regulation of JARID2 expression. [file 12885_2022_10263_MOESM5_ESM.pdf]

**A**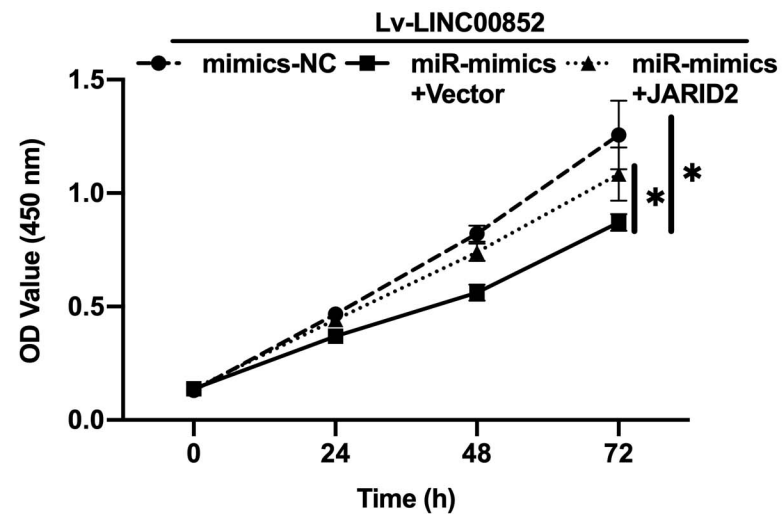**B**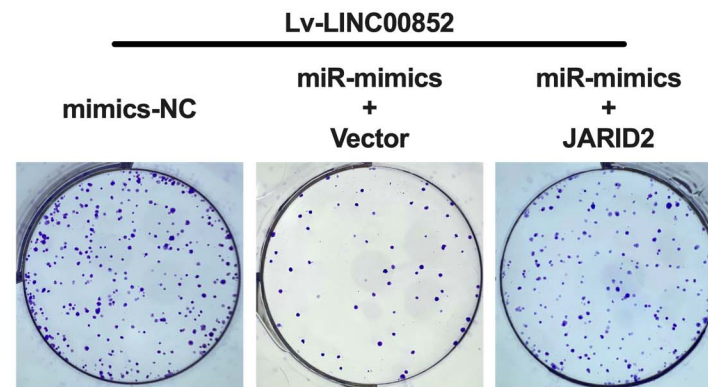**C**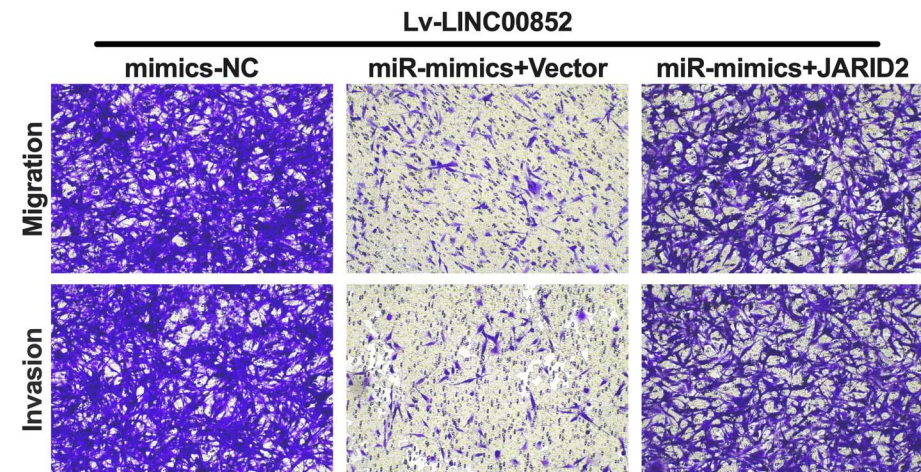**D**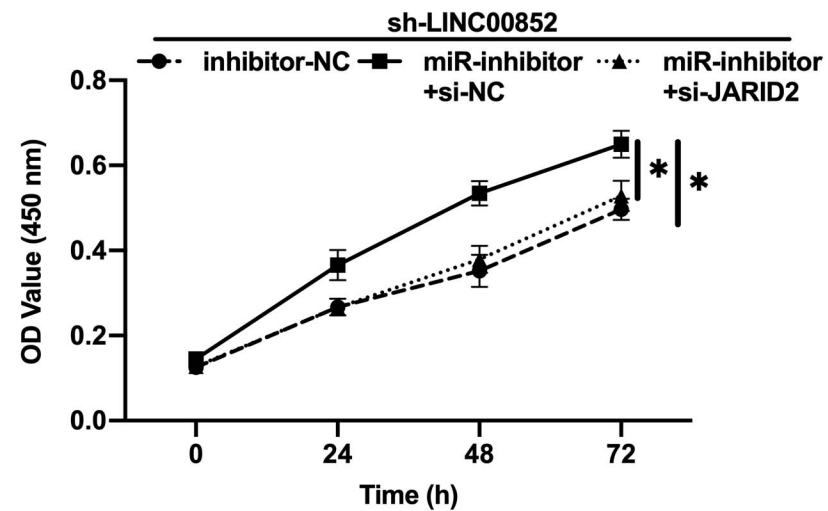**E**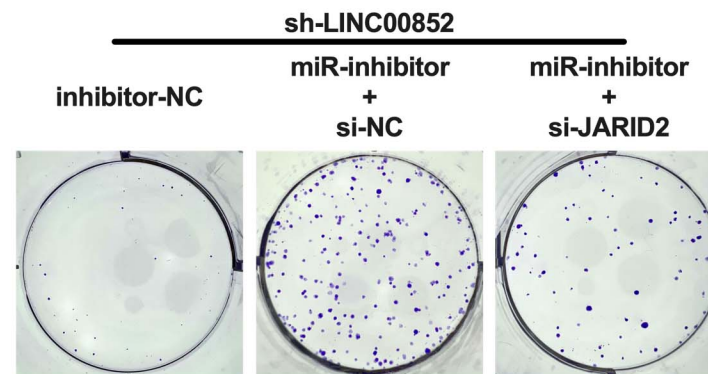**F**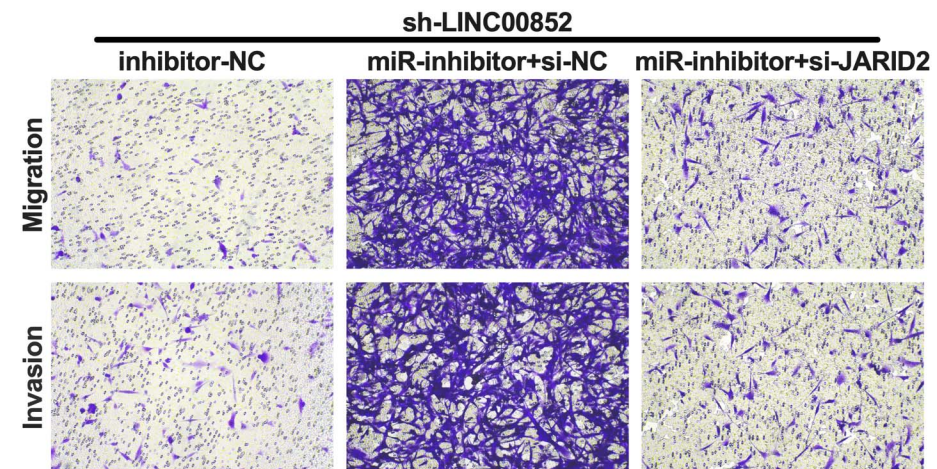

Supplement: Supplementary file 6 — Additional file 6: Figure S6. LINC00852 regulates the expression of JARID2 by targeting miR-29a-3p in PC-3 cells. [file 12885_2022_10263_MOESM6_ESM.pdf]

**A**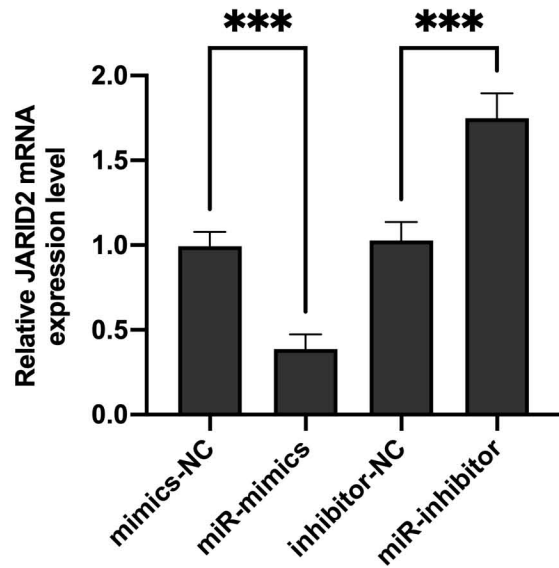**B**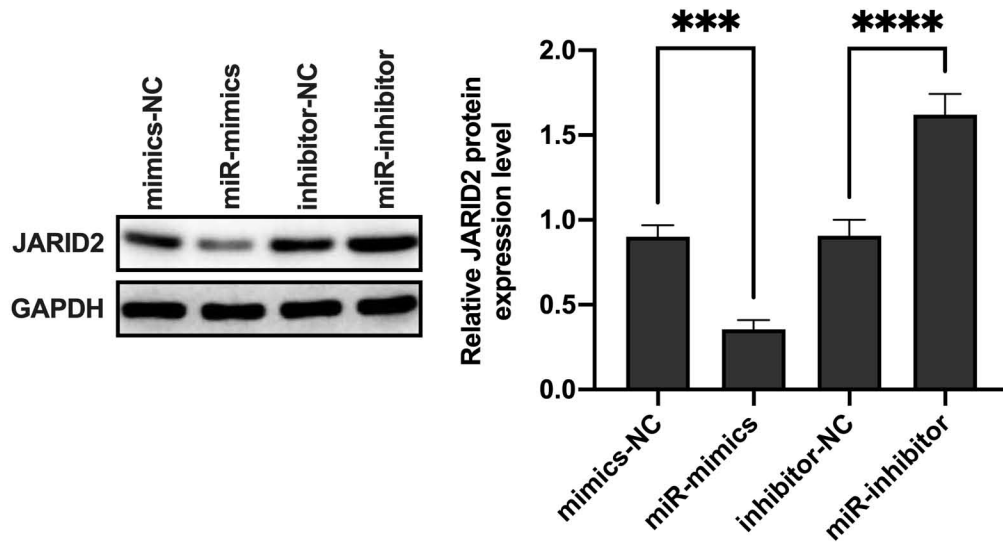

Supplement: Supplementary file 7 — Additional file 7: Figure S7. JARID2 regulated by miR-29a-3p in normal prostate epithelial cells RWPE1. [file 12885_2022_10263_MOESM7_ESM.pdf]
