# Supplementary material for: The LINC00852/miR-29a-3p/JARID2 axis regulates the proliferation and invasion of prostate cancer cell
Source: BMC Cancer. 2022 Dec 5;22:1269. doi: 10.1186/s12885-022-10263-6 (PMC9724404; doi:10.1186/s12885-022-10263-6)
Supplement: Supplementary file 8 — Additional file 8: Figure S8. Knockdown of LINC00852 increased xenograft tissues miR-29a-3p expression and decreased JARID2 mRNA expression in VCaP cells. [file 12885_2022_10263_MOESM8_ESM.pdf]

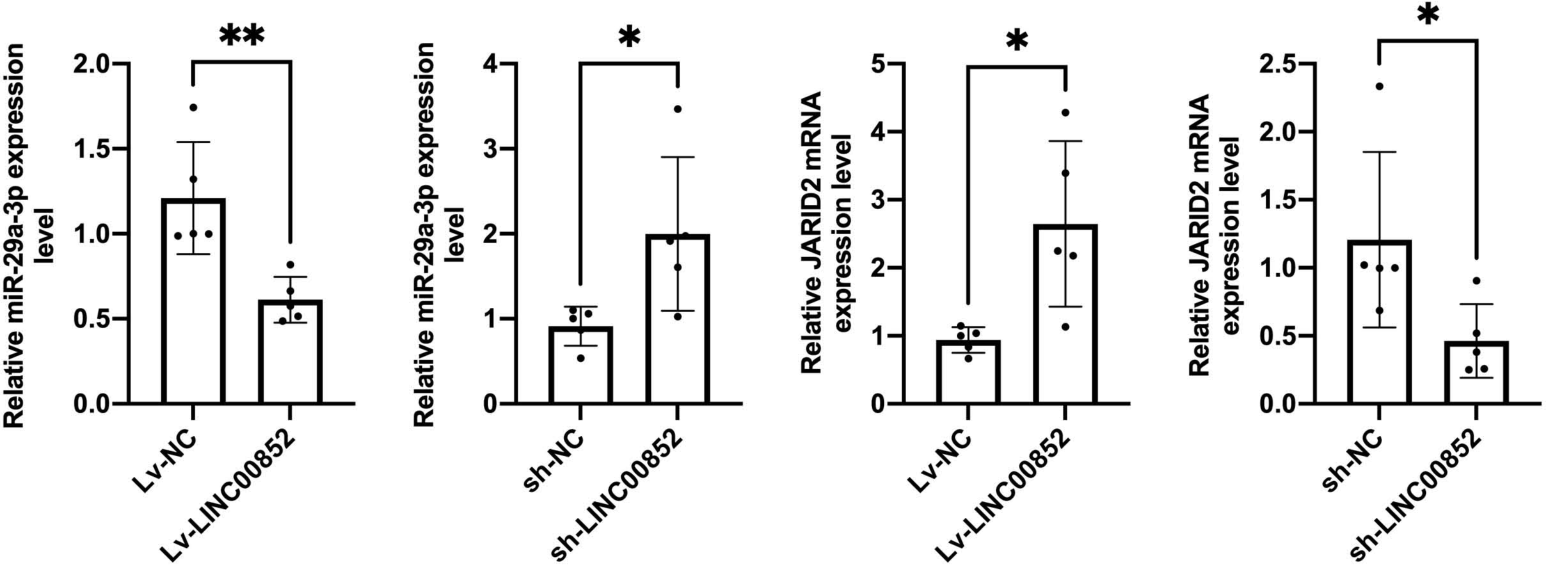
**
*
*
*
Relative miR-29a-3p expression level
Relative miR-29a-3p expression level
Relative JARID2 mRNA expression level
Relative JARID2 mRNA expression level
0.0
0.5
1.0
1.5
2.0
0
1
2
3
4
5
2.5
Lv-NC
Lv-LINC00852
sh-NC
sh-LINC00852
Lv-NC
Lv-LINC00852
sh-NC
sh-LINC00852
