# Supplementary material for: The LINC00852/miR-29a-3p/JARID2 axis regulates the proliferation and invasion of prostate cancer cell
Source: BMC Cancer. 2022 Dec 5;22:1269. doi: 10.1186/s12885-022-10263-6 (PMC9724404; doi:10.1186/s12885-022-10263-6)

FIG6D-GAPDH


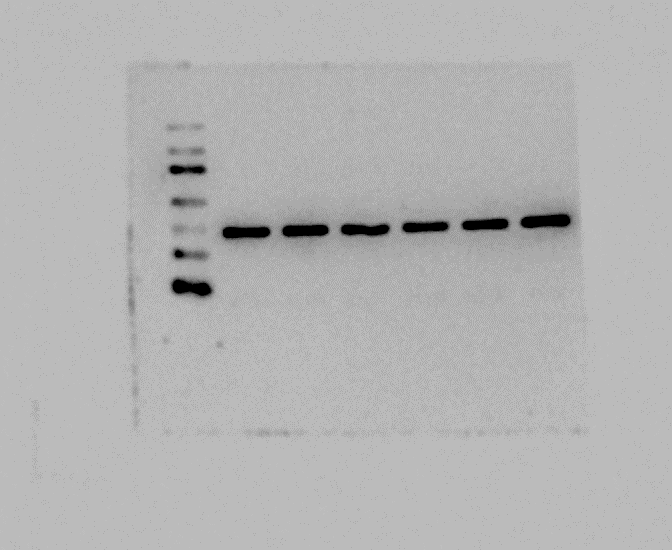


FIG6D-JARID2


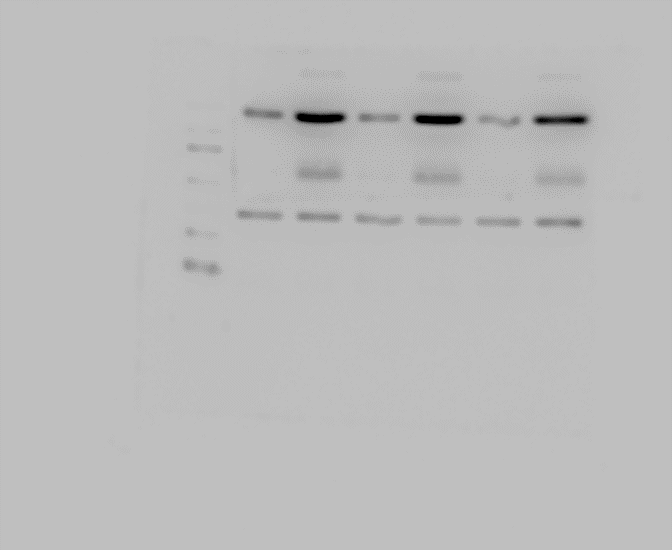


FIG6H-GAPDH


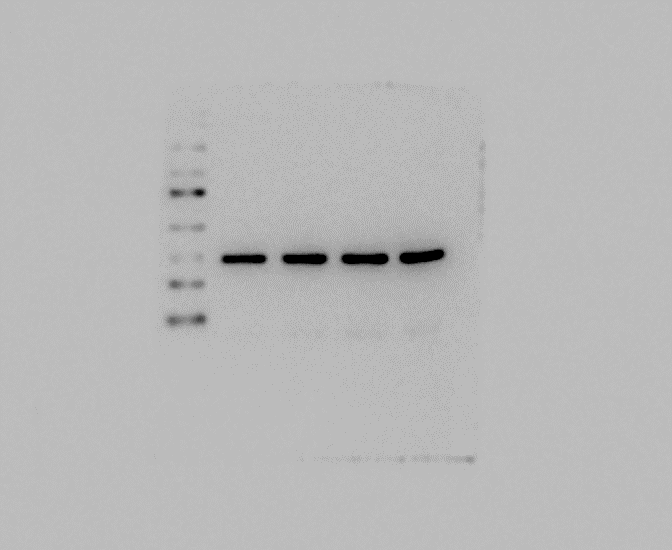


FIG6H-JARID2


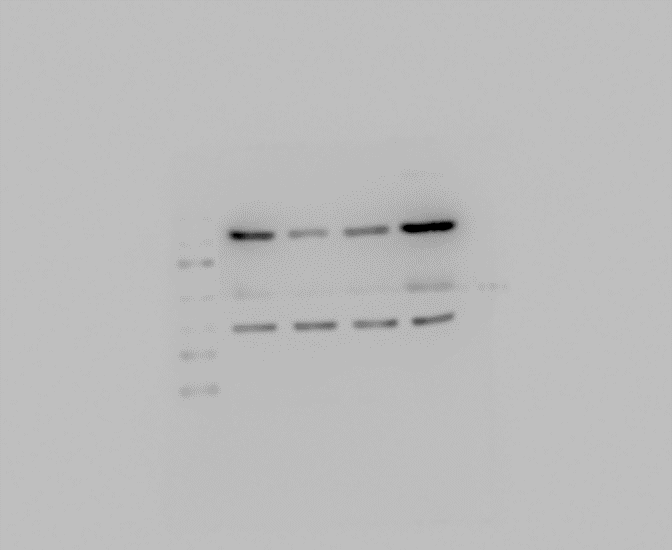


FIG6K-GAPDH


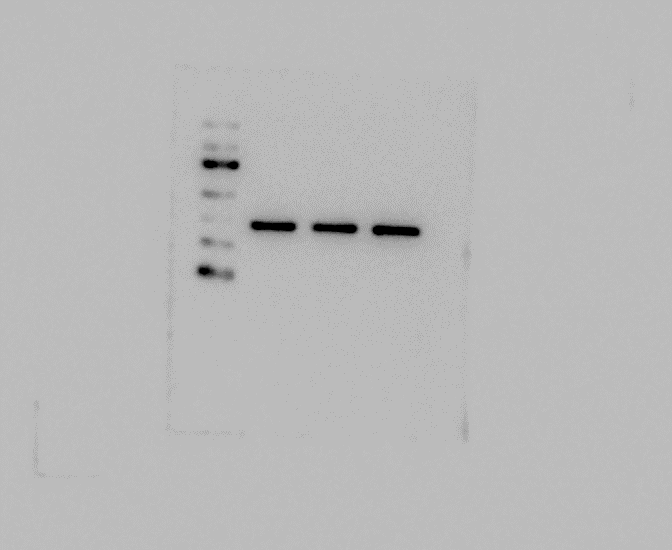


FIG6K-JARID2


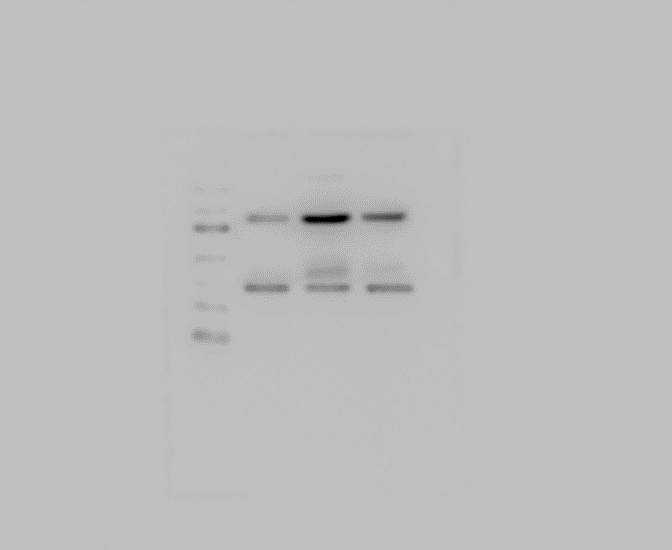


FIG6M-GAPDH


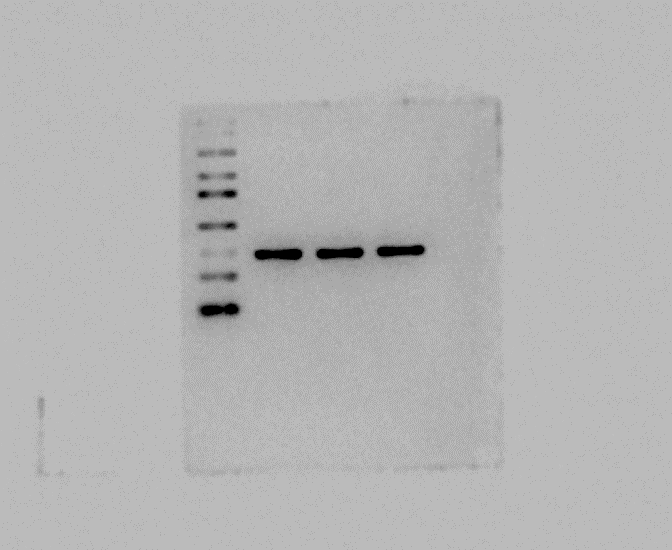


FIG6M-JARID2


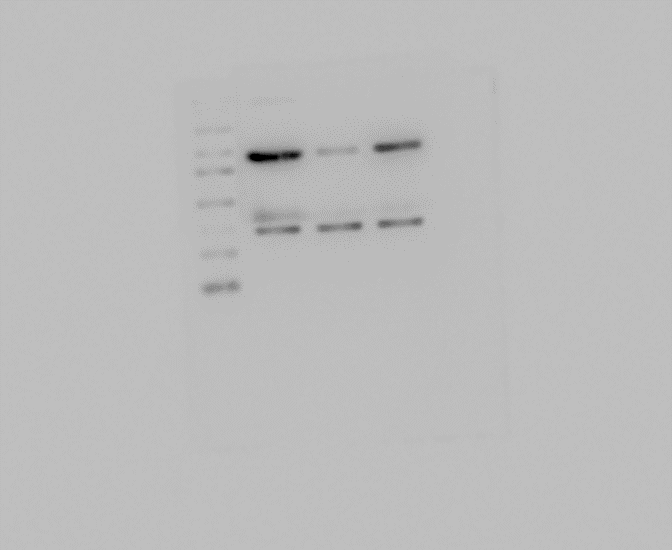


Supplement figure 4B GAPDH


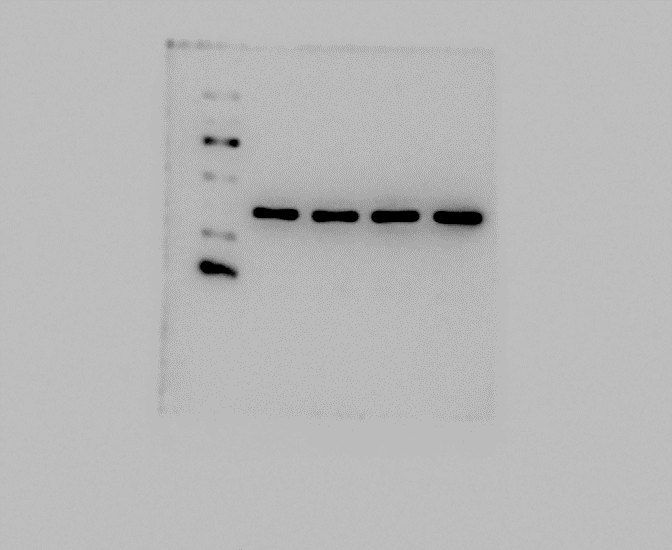


Supplement figure 4B JARID2


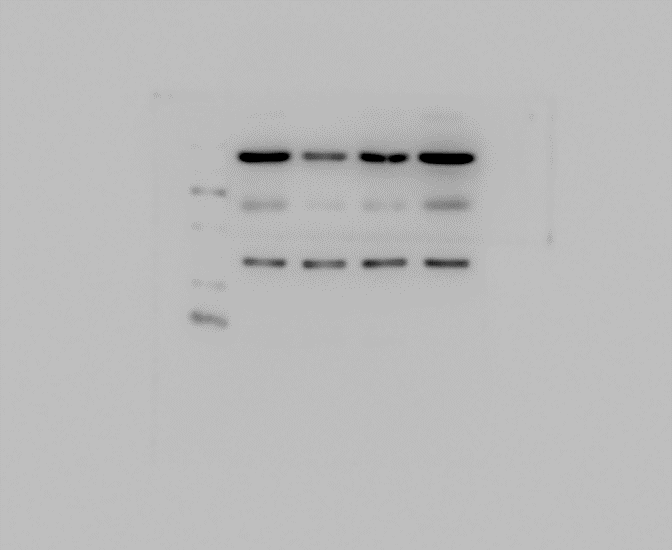


Supplement figure 4D GAPDH


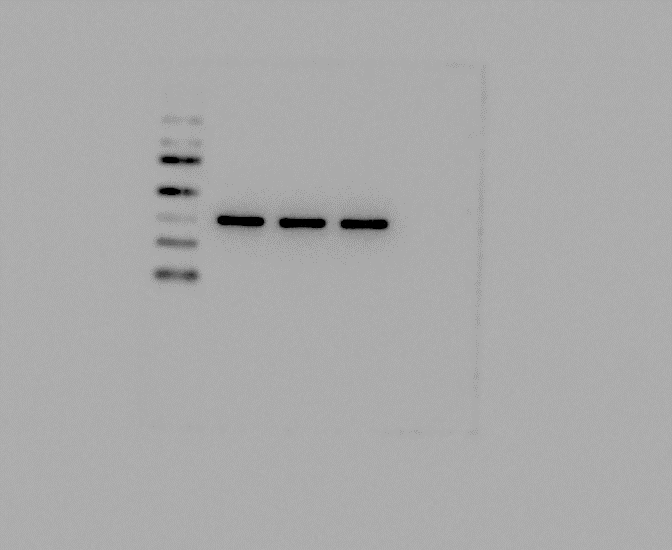


Supplement figure 4D JARID2


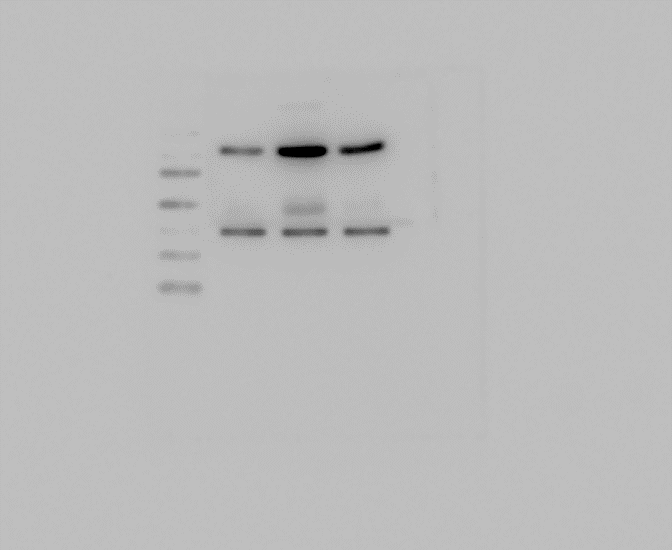


Supplement figure 4F GAPDH


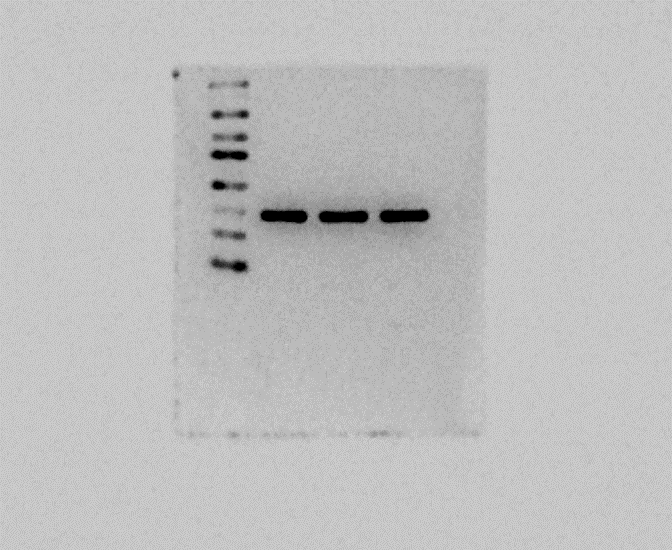


Supplement figure 4F JARID2


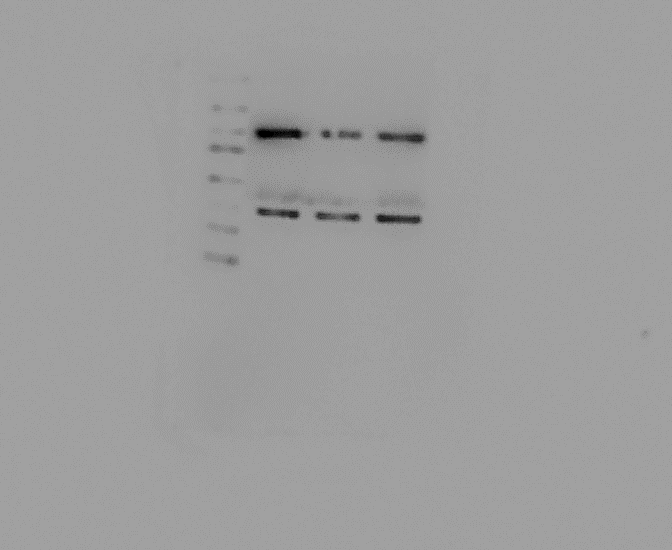


Supplement figure 7 GAPDH


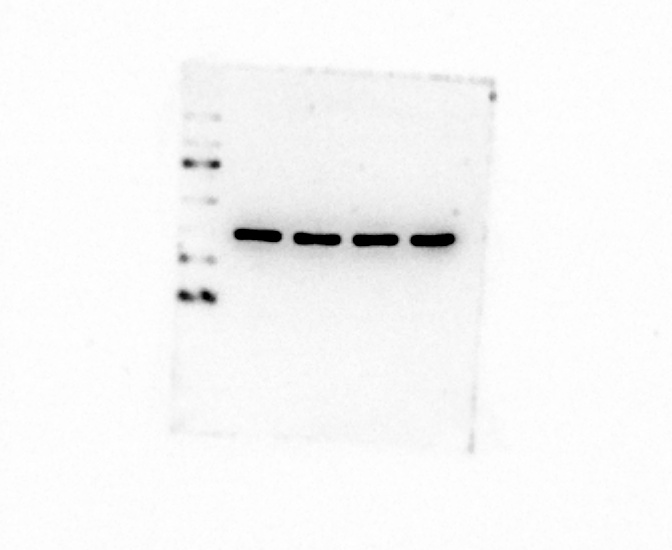


Supplement figure 7 JARID2


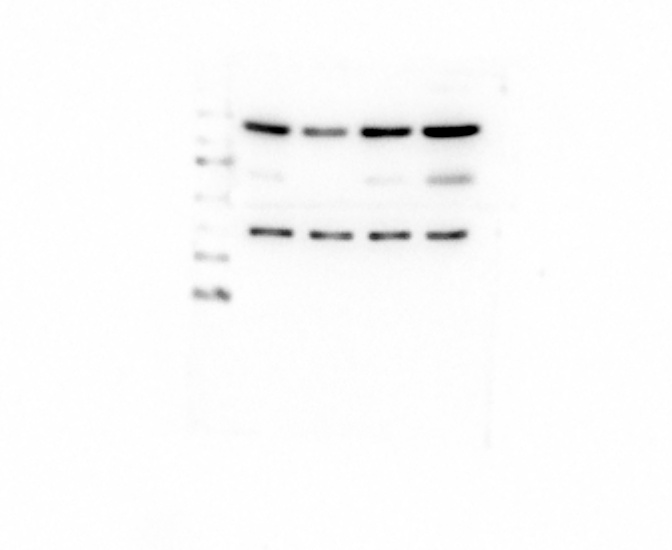

Supplement: Supplementary file 9 — Additional file 9: Original bands for Western blot. [file 12885_2022_10263_MOESM9_ESM.docx]
